# Dynamics of pesticide residues in soils during the growing season: a case study in peach orchards, east-central Portugal

**DOI:** 10.1007/s10661-025-13698-z

**Published:** 2025-02-13

**Authors:** Abel Veloso, Vera Silva, Rima Osman, Maria Paula Simões, Maria do Carmo Horta, Violette Geissen

**Affiliations:** 1 Soil Physics and Land Management Group, Wageningen University and Research, Wageningen, the Netherlands; 2 School of Agriculture, Polytechnic Institute of Castelo Branco, Castelo Branco, Portugal; 3 Research Centre for Natural Resources, Environment and Society (CERNAS), Castelo Branco, Portugal

**Keywords:** Peach orchards, Pesticide residues, Multi-residue analysis, Predicted versus measured environmental concentrations

## Abstract

Permanent crops rely heavily on pesticides. Their short-term behaviour has already been characterised under laboratory conditions. However, their degradation in the field is still poorly understood. Furthermore, studies that relate pesticide residues in soil with their application history are still scarce, despite their relevance for inferring the historical and diffuse dimensions of pollution. This work explores the spatial and temporal dynamics of pesticide residues in soil under the light of their application records and the accuracy of the predicted environmental concentrations (PEC). To achieve these purposes, 270 soil samples were collected at 5 sampling times over the 2022 growing season, in 18 peach orchards from east-central Portugal. Moreover, the data extracted from the application records was used to obtain predicted levels which were compared with the measured results. A total of 37 residues were analysed. Overall, 32 of these were detected back in, at least, one sample. Glyphosate and AMPA were the most frequently found and had the highest median concentrations. The comparison between predicted and measured environmental concentrations (MEC) suggests that pesticide decay was generally slower than expected. Glyphosate, AMPA, fluopyram and tebuconazole showed a high potential for resulting in background concentrations in soil. Our results highlight the need for regular and comprehensive monitoring, being recommended long-term assessments of the persistence and fate of pesticides under realistic conditions. Furthermore, our results also stress the necessity of more studies on reasons for PEC-MEC mismatch, a factor of concern considering the regulatory relevance of PEC values.

**Supplementary Information:**

The online version contains supplementary material available at 10.1007/s10661-025-13698-z.

## Introduction

Current agricultural systems are heavily dependent on pesticides to protect crops and increase yields (Oerke, 2006). From 2000 to 2021, world pesticide consumption grew from 2.2 × 10^9^ to 3.5 × 10^9^ kg.year^−1^, and application rates grew from 1.47 to 2.26 kg.ha^−1^. Although pesticide consumption in the EU has remained fairly constant over the last decade, around 3.5 × 10^8^ kg.year^−1^ (EUROSTAT, 2022; FAO, 2023), application rates have increased from 2.83 to 3.20 kg.ha^−1^ (FAO, 2023). Permanent crops, like vineyards and orchards, typically have a higher pesticide input than arable crops (Tona et al., 2018), which may result in increased pesticide residues in soil (Riedo et al., 2023; Silva et al., 2019).

Currently approved pesticide active substances (AS) are considered to be less harmful to non-target species and less persistent in the environment as compared to older ones that have been banned in most countries. However, out of the 441 AS currently allowed on the EU market, only 71 are classified as low risk (European Commission, n.d). Fifty AS are concerning due to their toxicity and persistence and are classified as candidates for substitution (European Commission, n.d.; Geissen et al., 2021; Hvězdová et al., 2018; Silva et al., 2019). The main concerns relate to the fact that (1) some effects are not direct and are only identified after long exposures; (2) the cumulative and combined effects of pesticides are difficult to assess; and (3) metabolites can be more toxic and persistent than their parent compounds (European Environment Agency, 2023; Hvězdová et al., 2018).

Concerns related to pesticides have been addressed by several initiatives. These compounds make up more than half of the substances listed by the Rotterdam and Stockholm conventions to be eliminated or severely restricted based on their health and/or environmental effects (Rotterdam Convention, 2010; Stockholm Convention, 2019). Within the EU, the sustainable use of pesticides regulation, in the line of the Farm to Fork strategy, aimed for a 50% reduction in pesticide use and risk by 2030. However, this regulation was rejected in 2023 by the European Parliament, and, in 2024, the European Commission proposed its withdrawal (Euronews, 2024).

Sixty percent to 70% of EU soils were assumed to be unhealthy (European Commission, 2023a). This problem is addressed by the EU Soil Strategy (ESS), adopted by the European Commission in 2021, and in more detail in the directive on soil monitoring and resilience, adopted in 2023 by the same commission as a proposal based on three main points: (1) monitoring soil health, (2) adopting sustainable soil management practices and (3) identifying contaminated sites. This directive aims to have all soils in a healthy condition by 2050, and pesticides are identified as one of the parameters to be evaluated. In Portugal, the PRoSolos law proposal on soil contamination established a framework to identify, monitor, and remediate contaminated sites (Participa, 2015). However, this law still has not been approved, leaving a significant gap in Portuguese soil legislation.

Soil is known as a sink and source for pesticide residues. Herbicides reach soils directly during application, while insecticides and fungicides (and other types of pesticides, used in smaller amounts) reach soils indirectly via interception from the crops (Gonçalves & Alpendurada, 2005; Riedo et al., 2023). Despite this recognition, and the recent legislative focus on soil and the sustainable use of pesticides, monitoring of pesticide residues in soils is not yet mandatory within the EU (European Commission, 2023a).

The inherent harmful characteristics of AS may affect ecosystems and humans. For example, 2,4-D and penconazole, both applied and analysed in this study, are suspected to be endocrine disruptors, and compounds from the neonicotinoids group, which includes acetamiprid, may not only be genotoxic but also negatively affect the liver and nervous system (European Environment Agency, 2023; Mnif et al., 2011; Peillex & Pelletier, 2020). Moreover, neonicotinoids may also be connected with the decline of insects, including pollinators. This fact alone justified the restrictions imposed on the use of clothianidin, imidacloprid and thiamethoxam, and eventually the non-renewal of the authorisations regarding these three AS. Despite this, emergency authorisations for the use of clothianidin, imidacloprid and thiamethoxam have been successively given by some member states (European Commission, 2023b). However, the European Commission is considering prohibiting all Member States from granting emergency authorisations (European Commission, 2023b; Foote, 2023).

An accurate prediction of pesticide residue concentrations in soil depends on the use of calibrated and validated approaches to calculate predicted environmental concentrations (PEC), based on application rates, adsorption and degradation over time. Unfortunately, pesticide fate is often complex and site-specific because it is affected not only by the intrinsic characteristics of pesticides but also by biotic and abiotic factors. Hydrophobic and positively charged compounds tend to bind more strongly to the soil matrix, especially to organic matter and clay, making them less available and less vulnerable to degradation by microorganisms (Gondar et al., 2013; Hvězdová et al., 2018; Masini & Abate, 2021). Pesticides may also affect the soil microbiome, which makes impact assessment often complex. For example, glyphosate negatively affects microorganisms that use the Shikimate pathway I for amino acid biosynthesis. As many soil-borne pathogens do not rely on this biochemical pathway, glyphosate may negatively impact the disease suppressiveness of soil (Geissen et al., 2021; Van Bruggen et al., 2018). The multitude of factors that affect pesticide degradation and the complexity of their interactions makes predicting residue concentrations over time increasingly difficult and highlights the necessity of monitoring pesticides in soil (Geissen et al., 2021; Hvězdová et al., 2018). This acquires an even higher relevancy if we consider that the approval (and ban) of a particular AS depends on those predictions (Geissen et al., 2021).

Peach orchards are an important crop in the east-central Portuguese region of Beira Interior, which is home to the country’s largest peach production area (Instituto Nacional de Estatística, 2019). However, as far as we know, the concentration of pesticide residues in soil has never been assessed for this crop or in this part of the country. This is a relevant knowledge gap, especially considering the need for monitoring soil health as expressed by the EU Soil Strategy, briefly described above (European Commission, 2023a). In addition to this, the heavy reliance of this crop on pesticides is also common for other permanent crops, particularly other orchards and vineyards, especially if we consider that the same AS are frequently used on different crops (Geissen et al., 2021; Silva et al., 2019; Tona et al., 2018). Therefore, the results from this study can serve not only to characterise the pesticide contamination status and decay in soils from the aforementioned region, but also as a stepping stone to other regions as well, especially those with similar edaphoclimatic conditions.

To address the afore mentioned knowledge gaps, this study looked into the pesticide residues in soils and their levels using peach orchards from east-central Portugal as case study sites. Soil samples were collected during the 2022 growing season, at 5 time points (i.e. February, April, June, August and October), in 18 orchards spread throughout the region. The information gleaned from the farmers’ application records was used to characterise pesticide applications in peach crops in this region and to predict residue concentrations in soils (EFSA, 2023; University of Hertfordshire, 2023). The main objectives of this study were (1) to characterise pesticide applications in the peach orchards (in terms of AS used and application rates), (2) to assess soil contamination status from pesticide residues and fluctuations in levels and composition of the pesticide mixture throughout the growing season (from February to October), and (3) to compare the predicted environmental concentrations (PEC), calculated using EFSA guidelines, with the measured environmental concentrations (MEC), evaluated during the growing season.

## Materials and methods

### Soil sampling

**Fig. 1:**
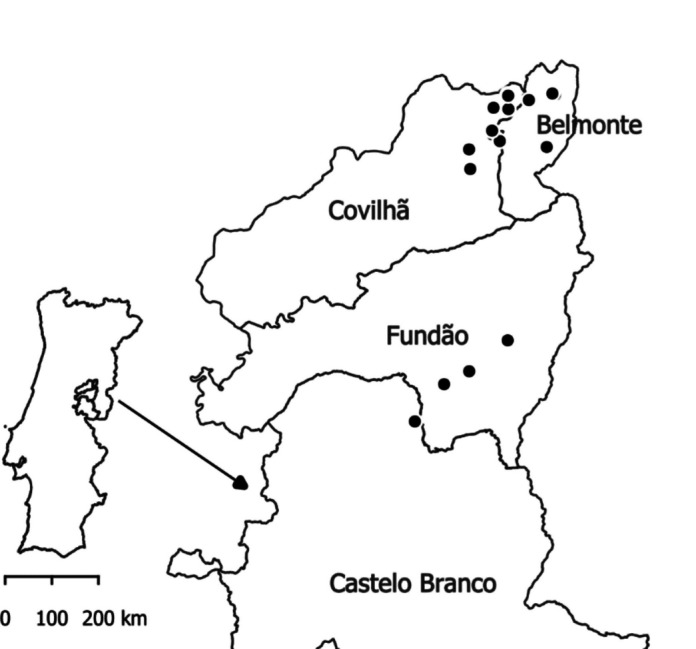
Distribution of the 18 peach orchards (represented as black dots) throughout the 4 municipalities covered in our study which are located in the central-east Portuguese region of Beira Interior. The data that is shown in the figure can be assessed at DGT (n.d.) and was organised with QGIS (version 3.28)

The soil samples used in this study were collected in February, April, June, August and October 2022. The sampling period and sampling frequency were chosen to cover most of the pesticide application moments, determined by the analysis of the pesticide records referring to 2022. These records were part of farmers’ field books and were provided to us by farmers and farmers’ associations as digital files. In some cases, these files included only information related to pesticide applications. In other cases, they also included information regarding other activities like irrigation and pruning. The soil samples originated from 18 peach orchards, whose areas ranged from 0.47 to 4.50 ha (median of 1.74 ha), located in the municipalities of Castelo Branco, Fundão, Covilhã and Belmonte, from the east-central Portuguese region of Beira Interior (Fig. 1). The Köppen-Geiger classification of the climate of this region is Csa (hot-summer Mediterranean climate), and the soils are mostly cambisols with a coarse texture and a mildly to moderately acidic pH (FAO, 2015; Kottek et al., 2006). For privacy reasons, the exact locations of the 18 orchards have not been provided. Instead, each orchard was identified by a code (F01, F02, …, F18). All orchards were managed under the same basic principles and were planted either in 2018 or in 2019. The weeds in the rows are controlled by herbicide applications as no tillage is conducted after planting. In the inter-row space, weeds are mowed periodically. Drip irrigation is used in all orchards. The water comes either from farm based rainfed ponds or from local dams.

Three composite samples were collected per orchard using a soil auger (field replicates). Considering that the shape and size of the orchards were variable and in order to maximise the representativeness of the samples, each composite sample was collected in each of the three tree rows that were obtained after dividing the orchard in four parts (Figure S1, Supplementary Material). Each composite sample was formed by mixing five subsamples, evenly spread throughout one row of trees. Samples were collected from approximately the same place for every sampling time. Considering that most pesticides accumulate in the top soil layer (Laitinen et al., 2006; Yang et al., 2015) and following the guidelines of the EFSA (2015) for permanent crop assessments, the chosen sampling depth was 0–5 cm. After collection, the samples were air dried in the dark for a period of no longer than 1 week (until their moisture content was below 4%) and then sieved (2 mm) and stored at − 20°C until pesticide analysis.

The collection of three mixed samples per orchard, in 18 orchards and 5 time points resulted in a total of 270 samples. All of these were analysed. The average pesticide content from the 3 samples per orchard per time point was determined, resulting in 90 results per compound, orchard and time point reported below. The collection of three composite samples per orchard and time point allowed to compensate, at least partially, for the spatial variation in pesticide concentrations. In fact, the coefficient of variation, i.e. the ratio between the standard deviation and the average of the three samples/orchard/time point, varied between 0.704 and 140%.

### Selection of analytes, pesticide analysis and quality control

The list of pesticide residues to analyse was based on the pesticide application records provided by the local farmers and farmers’ associations. To that list, the major metabolites and a few other commonly found AS of environmental concern were added.

Due to analytical and financial limitations, some substances listed in the records were not analysed. These included captan, emamectin, lambda-cyalothrin, paraffin oil, tau-fluvalinate and ziram. In some cases, like captan, tau-fluvalinate and ziram, this decision was also supported by their low persistence, expressed by their low DT_50_ values, i.e. 0.8 days, 1.62 days and 4 days, respectively (University of Hertfordshire, 2023). Inorganic pesticides, such as copper salts and sulphur, were also not analysed. Preference was given to substances that could be extracted using a multi-residue method, which followed the methodology of Anastassiades et al. (2003) and Silva et al. (2019). Due to their environmental importance, glyphosate and aminomethylphosphonic acid (AMPA) were exceptions to this and were extracted separately, following the methodology of Yang et al. (2015) and Silva et al. (2019). In the end, a total of 28 AS (23 compounds present in records and 5 considered as relevant) and 9 metabolites were tested in the soil samples. The limits of detection (LOD) and quantification (LOQ) of these residues ranged between 0.1 and 10.0 µg.kg^−1^ and between 1.0 and 25.0 µg.kg^−1^, respectively (Table S1, Supplementary material).

Soil samples were thawed 1 day before pesticide analysis was carried out. After homogenisation, two aliquots were taken and added to 50-mL Greiner tubes, one aliquot of 5 g for the multi-residue method and the other of 2 g for the determination of glyphosate and AMPA.

Regarding the multi-residue method, each sample was spiked with 25 µL of 10 µg.mL^−1 13^C_3_-caffeine (used as a surrogate to evaluate the efficiency of all steps within a procedure of LC–MS/MS). After that, 5 mL of Millipore water and 10 mL of acetonitrile in 1% acetic acid were added. The tube was then agitated end-to-end for 30 min, and 1 g of sodium acetate and 4 g of magnesium sulphate were added. After the tube was vortexed and centrifuged (5 min at 3500 rpm), 250 µL of the supernatant was transferred to a liquid chromatography (LC) filter vial, and 250 µL of Millipore water was added.

For the glyphosate and AMPA extraction, 50 µL of the internal standard solution (5.0 µg.mL^−1^) and 10 mL of 0.6 M potassium hydroxide were added to a 50-mL Greiner tube containing the 2 g soil sample. The tube was agitated end-to-end for 60 min, centrifuged for 10 min at 3500 rpm, and 1 mL of the supernatant was transferred to a 10-mL centrifuge tube. To this tube, 80 µL of 6 M hydrochloric acid, 0.5 mL of borate 5% buffer and 0.5 mL of 6.5 mM 9-fluoroenylmethoxycarbonyl chloride (FMOC-Cl) were added. The tube was vortexed for 15 s and left for 30 min. After that, 50 µL of 98–100% formic acid was added, the tube was vortexed again, and 0.5 mL of the extract was transferred into a LC filter vial.

The quality control of both methods followed the SANTE guidance document (European Commission, 2017). The calibration standards for LC–MS/MS analysis were prepared using a solution of 1 part acetonitrile in 1% acetic acid and 1 part Millipore water as a solvent for the multi-pesticide extraction and Millipore water for the glyphosate and AMPA extraction. For the multi-pesticide analysis, the calibration curve was plotted using the following standards: 0.1, 0.5, 1, 5, 10, 50, 100 and 150 ng.mL^−1^. The calibration curve used in the glyphosate/AMPA analysis was based on the following standards: 1, 2, 5, 10, 25 and 100 ng.mL^−1^. The calibration standards were injected at the beginning of the sample sequence. All calibration curves showed correlation coefficients higher than 0.99 with a deviation of back calculated concentration lower than ± 20%.

For quality assurance and quality controls, each sequence of samples included blank soils (soils without the tested pesticide residues) spiked with the mixed solutions of the reference standards. For the multi-residue analysis, four spiked concentrations were used (0.5, 1, 5 and 10 µg.kg^−1^), and for the glyphosate-AMPA analysis, two spiked concentrations were used (10 and 50 µg.kg^−1^). The recovery rates were between 70 and 120%.

The identification of analytes was based on the retention time and peak shape of the reference standard (or isotopically labelled standard for glyphosate and AMPA) and on the ion ratio. For the ion ratio, the ratios between the quantification and confirmation transitions were within the interval of ± 30% of the average ion ratio of the calibration standards.

In the multi-residue analysis, the average of the concentration of all standards in the solvent (5 ng.mL^−1^), analysed every nine sample injections, was used to calculate the concentration of the analytes. In the glyphosate-AMPA analysis, the concentration of the analytes was determined based on bracketing the calibration with a standard containing labelled glyphosate and AMPA at 10 ng.mL^−1^, which was analysed every nine to ten injections. Glyphosate and AMPA responses were normalised according to the response of the isotopically labelled analogues.

The limits of quantification were determined using the lowest concentration of the spiked analyte that met the requirements defined by the European Commission (2017). The limits of detection were estimated using the blank soil samples with the lowest concentrations and a signal to noise ratio of 3.

### Data analysis

Compounds whose content was below the LOD were considered as not present in the sample. Those cases were, therefore, ignored in the subsequent analysis. However, compounds whose content was equal or above the LOD but below the LOQ were considered present and, as such, accounted for in the number of residues in the sample, in the applied-detected analysis and in the frequency of detection calculations. Nevertheless, they were not accounted for in the concentration-related indicators, in the average of replicates or in the total pesticide concentration/sample. On the one hand, setting a unique LOD value (and, as such, common to all compounds) would make comparisons between compounds more reliable. On the other hand, it would also imply that some compounds would be ignored even if, in some cases, their concentrations were high enough to be detected. Considering all of this, we decided to use the LOD for each compound which was determined based on the analytical technique explained above.

PECs were determined at the orchard level based on each application scheme using the software Escape, version 2.0 (Fraunhofer IME, Germany). This software allows the input of more than 1 application event. Considering this, in the cases where the AS was applied more than once, each application event was included in the software before it was run to determine the PEC. The output from this software gives the predicted AS concentrations (PECs) at several timepoints. From this list, only the compounds which had a corresponding MEC to be compared with were selected. When applicable, PECs of the metabolites were calculated in the same round as the parent compound. The kinetic models used for the PECs determination for each compound are presented in Table S2 (Supplementary Material), and their equations can be found in EFSA (2006).

The application date(s) and rate(s) of application of each AS were obtained from the application records provided by the farmers (Table S3, Supplementary material). This approach was the same as that used in the EFSA dossiers for the approval of AS. For this reason, the generic bulk density of 1.5 g/cm^3^ was used, and crop interception was set to 0% for herbicides and 70% for the other AS (acaricides/insecticides and fungicides). The kinetic parameters, including the half-life time in soil (DT_50_), were obtained from the aforementioned EFSA dossiers or, in the few cases when not found there, from the Pesticide Properties Database/PPDB (University of Hertfordshire, 2023). PPDB typical half-life times were preferred over lab or field-based entries.

The list of compounds present in farmers’ records was compared with the list of compounds detected in the soil. This analysis was performed at orchard level using an approach similar to what was presented by Riedo et al. (2023). The comparisons between applications and findings were divided into one of the following categories: (1) applied and detected, (2) applied but not detected, (3) not applied but detected, and (4) not applied and not detected. The last category included the AS that, although not applied in any of the orchards, were considered relevant. This analysis took into consideration the application time defined in farmers’ records. For example, an AS detected in a sample collected before its first application of 2022 was classified as “not applied but detected”.

For the comparison of PEC and MEC, only MEC values equal to or higher than the respective LOQ were considered. The other MEC values, i.e. those which were below LOQ, were not set to 0. Instead, they were removed from the spreadsheet. As PEC determination was not experimentally based, and thus not subjected to the LOQ, all PEC values were considered, providing that there was a corresponding MEC to be compared with. However, it is worth noting that not considering the cases where MEC values were below LOQ implied a reduction in the PEC-MEC comparisons, including those for which both parameters were below LOQ.

DT_50_ values were also used to classify the persistence of the AS and the metabolites into three categories: low (DT_50_ < 30 days), medium (30 days ≤ DT_50_ ≤ 100 days) and high (DT_50_ > 100 days), adapted from PPDB, Silva et al. (2019) and Riedo et al. (2023).

The statistical analysis and data visualisation were performed with R (version 4.3.1). The normal distribution and homogeneity of variances of the total pesticide content were tested using Shapiro–Wilk’s and Levene’s tests, respectively, the latter from the “car” package (Fox & Weisberg, 2019). As the data did not follow a normal distribution, differences in total pesticide content between sampling months were tested using the non-parametric Kruskal–Wallis’ test. The significance level was set at 0.05. Data visualisation, including the heat maps, was performed using the “ggplot2” package (Wickham, 2016).

## Results and discussion

### Pesticide applications

According to farmers’ records, 31 different active substances (AS) were applied in the 18 selected peach orchards during 2022 (Table S3, Supplementary material). From these substances, 45.2% were fungicides, 41.9% were insecticides and/or acaricides, and 12.9% were herbicides. Approximately 29% of the AS that were reported to have been applied were of high concern to the EU as they are classified as candidates for substitution and, therefore, are intended to be withdrawn as soon as viable alternatives are found (European Commission, n.d.).

The number of different AS applied per orchard varied between 5 (in orchard F14) and 15 (in orchard F05), with a median of 7. The range of values were lower than what was reported by Chiaia-Hernandez et al. (2017) and by Riedo et al. (2023) for orchards in Switzerland and higher than the range presented by Geissen et al. (2021) for orange orchards in Spain.

The total application of AS per orchard varied between 4160 g.(ha.a)^−1^ (in orchard F14) and 29,204 g.(ha.a) ^−1^ (in orchard F05), with a median and standard deviation of 14,317 ± 7413 g.(ha.a)^−1^, similar to what Chiaia-Hernandez et al. (2017) found in Swiss orchards. The number of pesticide applications per orchard in 2022 ranged from 7 (in orchard F14) to 26 (in orchard F05) with a median of 11.5.

**Fig. 2:**
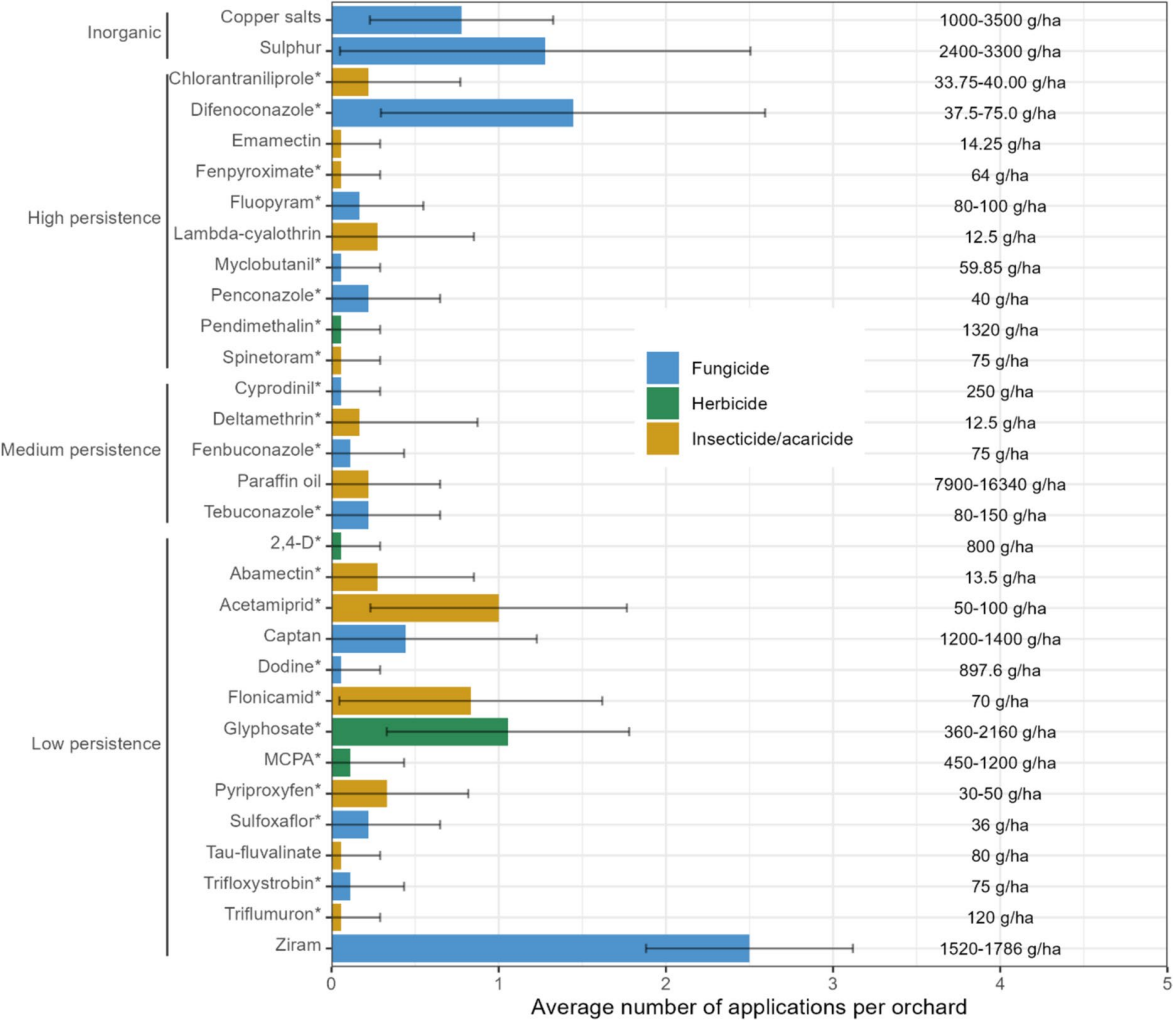
Pesticide use intensity in the peach orchards. The bars indicate the average number of applications per orchard for each active substance (AS), according to farmers’ records, in the 18 peach orchards from the east-central Portuguese region of Beira Interior during 2022. AS were dived into three categories, based on their DT_50_ values. More precisely, the persistence of an AS was considered to be low if its DT_50_ was less than 30 days, medium if its DT_50_ was between 30 and 100 days and high if its DT_50_ was more than 100 days. A 4th category was included to accommodate the two inorganic AS that were also reported to have been applied in the orchards. The labels on the right of each bar indicate the application rate (single value or interval) of each AS applied in the orchards. AS that were analysed appear in the plot marked with a *

The 5 most frequently applied AS across the 18 orchards (Fig. 2) during 2022 were ziram (2.5 ± 0.6 applications per orchard in 2022), difenoconazole (1.4 ± 1.1 applications per orchard in 2022), sulphur (1.3 ± 1.2 applications per orchard in 2022), glyphosate (1.1 ± 0.7 applications per orchard in 2022) and acetamiprid (1.0 ± 0.8 applications per orchard in 2022). The application rates of AS varied between 12.5 g.ha^−1^ (for deltamethrin) and 16,340 g.ha^−1^ (for paraffin oil).

### Pesticide residues in soil

**Fig. 3:**
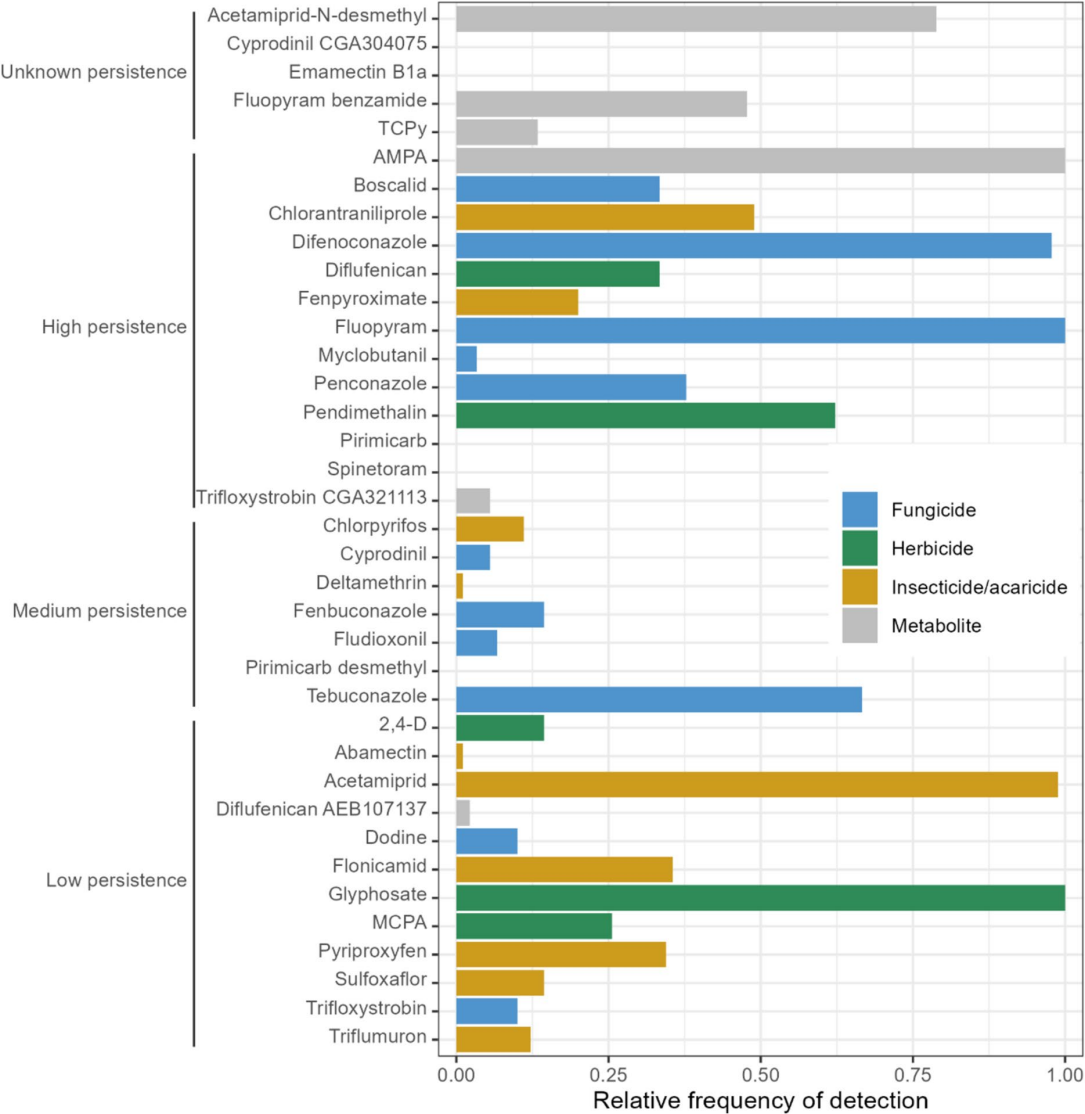
Frequency of detection of the active substances and metabolites that were analysed from all the soil samples collected in 2022 in peach orchards from the east-central Portuguese region of Beira Interior. *N* = 90 results for each active substance/metabolite. Active substances and metabolites were dived into three categories based on their DT_50_ values. More precisely, the persistence was considered to be low if the DT_50_ was less than 30 days, medium if the DT_50_ was between 30 and 100 days and high if the DT_50_ was more than 100 days. A 4th category included the active substances and metabolites whose DT_50_ values were not found in literature

As shown in Fig. 3, the five most frequently detected residues were the herbicide glyphosate (in 100% of the orchards and sampling months) and its main metabolite AMPA (100%), the fungicide fluopyram (100%), the insecticide acetamiprid (98.9%) and the fungicide difenoconazole (97.8%).

Glyphosate and its main metabolite, AMPA, have been reported in literature as two of the most frequently found compounds (Geissen et al., 2021; Riedo et al., 2023; Silva et al., 2019). Unlike difenoconazole, fluopyram and acetamiprid are not so commonly referred in bibliography. However, the fungicide fluopyram was one of the most commonly detected pesticides by Froger et al. (2023) in French soils (0–20 cm depth) under several land uses. Regarding conazole fungicides, Beriot et al. (2023) found, among other AS, difenoconazole at a high frequency with concentrations higher than 1 µg.kg^−1^ in samples collected in vegetable farms in Spain (0–10 and 10–30 cm depth), and Kosubová et al. (2020) found conazole fungicides in all of their samples of arable soil from the Czech Republic (0–25 cm depth). It is worth noting that conazole fungicides, like difenoconazole and tebuconazole, are listed as candidates for substitution by the Regulation (EU) 2015/408 because of their persistency and toxicity (European Commission, 2015).

Although the use of acetamiprid is still allowed within the EU, other neonicotinoids like imidacloprid have already been banned mainly because of their toxicity to pollinators (European Commission, 2023a). Nevertheless, despite imidacloprid being banned in the EU since 2018, it can still be found in soil (e.g. Beriot et al., 2023; Geissen et al., 2021).

High half-life (DT_50_) values do not thoroughly explain these high frequencies of detection. In fact, although the DT_50_ values of AMPA, fluopyram and difenoconazole are high (633 days, 309 days and 265 days, respectively), this is not the case for the DT_50_ of glyphosate (16.11 days) nor acetamiprid (12.96 days), which suggests that their persistency is higher than expected.

**Fig. 4:**
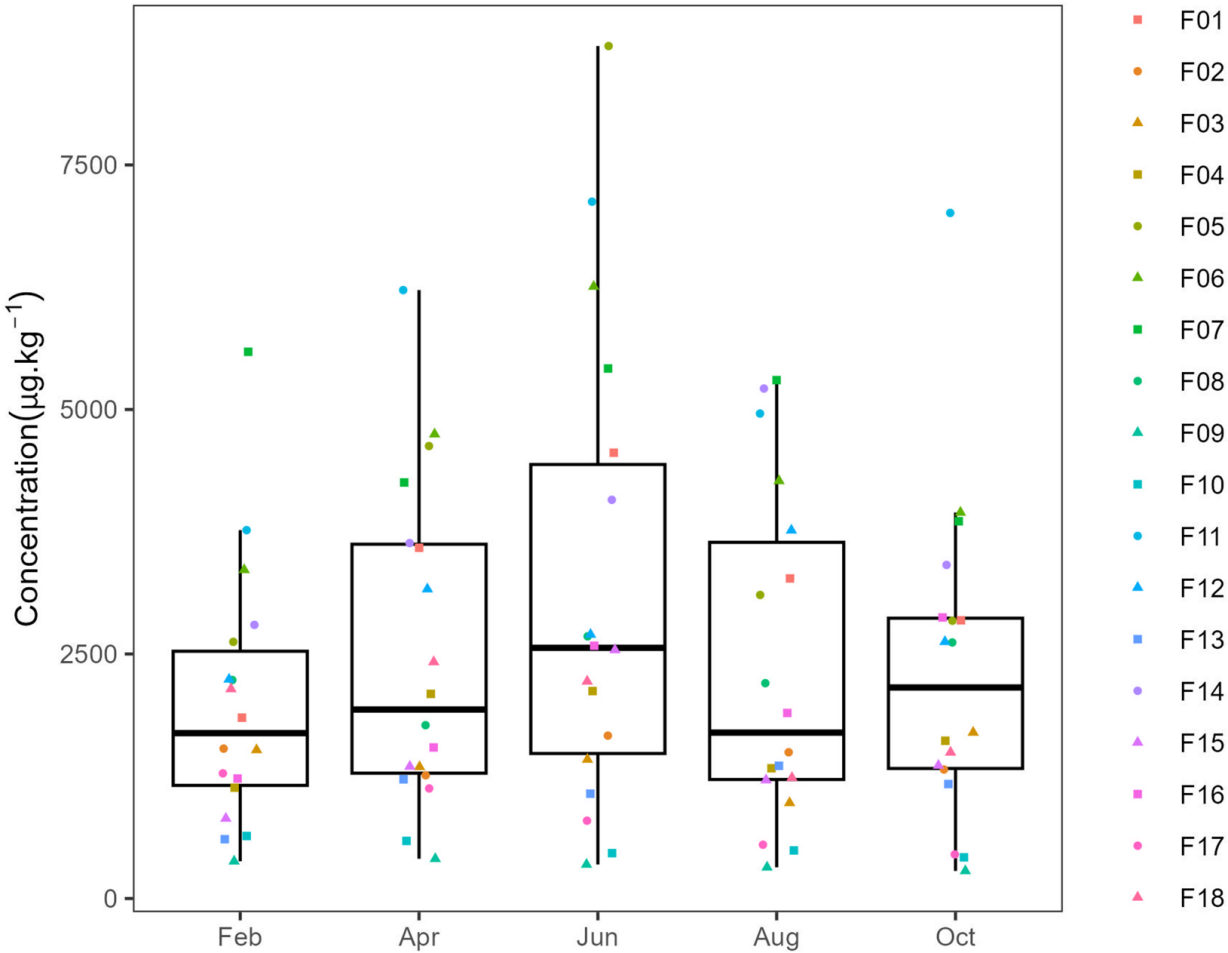
Boxplots representing the total pesticide concentrations for each sampling month in the samples collected in 2022 from 18 peach orchards in the east-central Portuguese region of Beira Interior. No statistical differences were found between sampling months (*p* ≥ 0.05) by the non-parametric Kruskal–Wallis test. *N* = 18 results for each month indicated in the plot. In each boxplot, the boxes represent the 1st, 2nd/median and 3rd quartiles, the whiskers represent 1.5 of the inter-quartile length or minimum/maximum, and the dots represent the individual values

Pesticide residues were found in all orchards and sampling months. The number of AS and metabolites detected varied between 3 (orchard F16, in February) and 15 (orchard F04, in June and August), with a median of 7. The total pesticide content (i.e. the sum of all residues in the sample) ranged from 282.1 µg.kg^−1^ (orchard F09, in October) to 8716.2 µg.kg^−1^ (orchard F05, in June) (Fig. 4).

The median values of the total pesticide content (AS and metabolites) ranged between 1691 µg.kg^−1^ in February and 2563 µg.kg^−1^ in June (Fig. 4). No statistical differences between sampling months (*p* ≥ 0.05) were found.

Both the maximum and the median of total content values were lower than what was found by Geissen et al. (2021) but higher than what was reported by Hvězdová et al. (2018), Riedo et al. (2022) and Silva et al. (2019). The last authors reported the highest pesticide content in soils from Portuguese vineyards with median and maximum values of, respectively, 1990 and 2870 µg.kg^−1^, mostly due to glyphosate and AMPA, and the soils from permanent crops with the highest pesticide content and the highest frequency in soils with pesticide content above 1000 µg.kg^−1^. Nevertheless, it is worth noting that comparisons between studies should be considered with caution because there are factors like the sampling method (including depth of sampling), sampling time, field conditions, laboratory methodologies and lists of analytes that may differ. In particular, sampling depth may be of great importance as the concentration of pesticide residues is often higher at the soil surface (Gonçalves & Alpendurada, 2005; Laitinen et al., 2006; Silva et al., 2019; Yang et al., 2015). In line with this, it should be added that, in all of the aforementioned studies, the sampling depth was deeper than in ours (i.e. 0–5 cm), varying between 0–10 cm (Riedo et al., 2022) and 0–25 cm (Hvězdová et al., 2018). The number of analytes per sample is also variable between the aforementioned studies, ranging from 46 molecules (Hvězdová et al., 2018) to 76 molecules (Silva et al., 2019).

The total content presented above does not represent the real total pesticide content but the sum of the content of all compounds that were analysed in a given sample and whose result was higher than LOQ. Therefore, the real value would be at best equal to but more likely higher than this parameter presented not only in our study but also in others as well (Geissen et al., 2021).

Glyphosate and AMPA were the residues found in the highest levels by far in soil (with median concentrations across sampling times of 584.1 µg.kg^−1^ and 1058.9 µg.kg^−1^, respectively; Table S1, Supplementary material) and maximum values of 4542.6 µg.kg^−1^ and 4649.3 µg.kg^−1^, respectively. Two potential reasons for this could be that (1) applications of glyphosate occurred shortly before the sampling times and (2) application rates were particularly high. However, neither of the reasons conveniently explains the high concentrations of glyphosate and, therefore, its main metabolite (AMPA) in soil. In fact, glyphosate was applied in most orchards in March (Table S3, Supplementary material), while the next samples were collected at the end of April. However, the application rates of glyphosate were among the highest which may partially explain its high concentrations in soil. Nevertheless, they were not the highest, and, more importantly, glyphosate concentrations in soil were already high in the February samples, which were collected before the first glyphosate application of the year and, probably, several months after the last application. Glyphosate is usually applied in the orchards during late winter or early spring. This suggests that the slow decay rate of glyphosate and AMPA should be the most important factor to explain their high concentrations in soil. This subject will be developed further below. However, it is worth noting that other studies also refer the presence of pesticide residues in soil long after their application (e.g. Chiaia-Hernandez et al., 2017; Froger et al., 2023).

### AS applications based on farmers’ records from 2022 and residue detection in soils

**Fig. 5:**
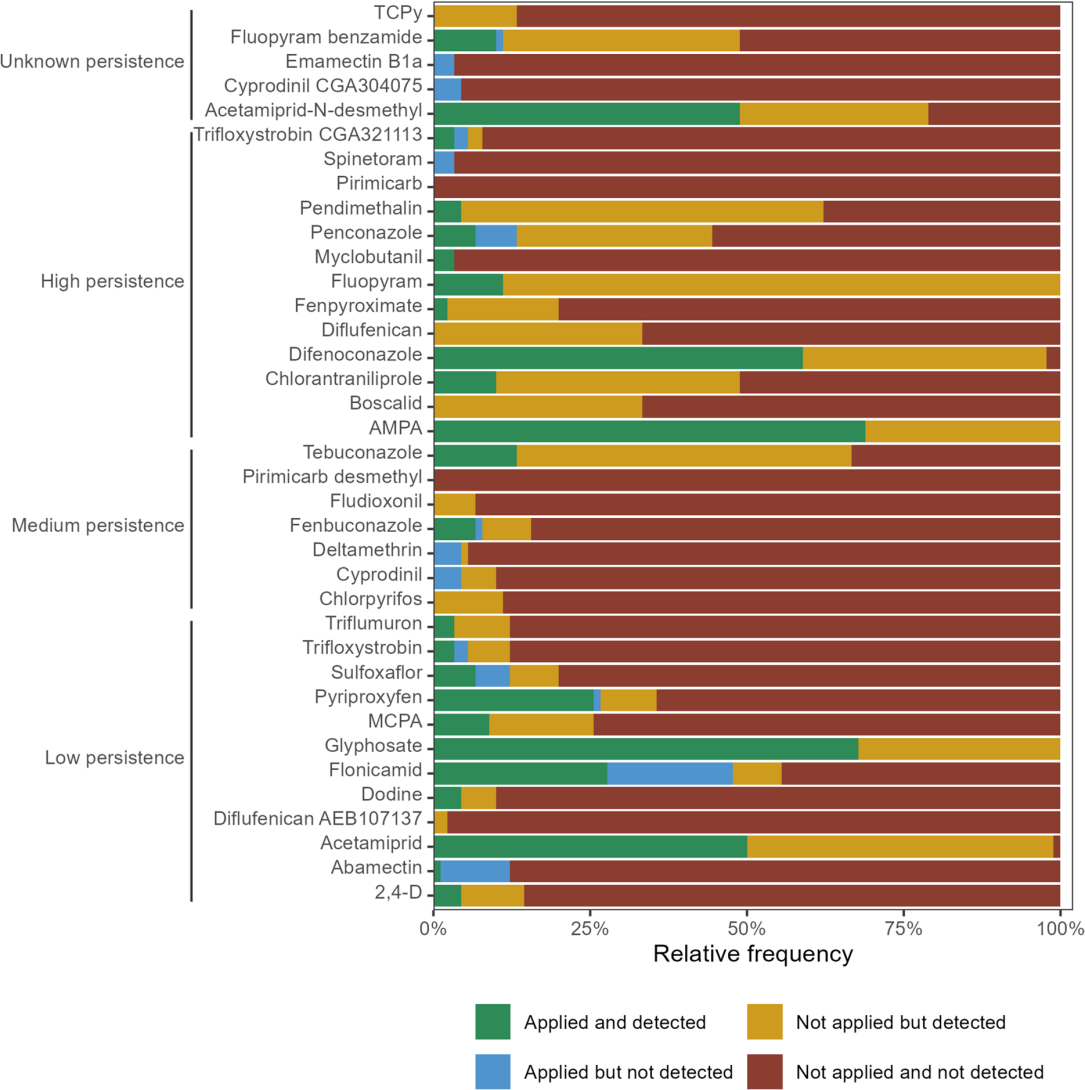
Comparison between pesticide application and detection for active substances and metabolites. The plots show the relative frequency distribution of the results divided into four categories: applied and detected, applied but not detected, not applied but detected and not applied and not detected. *N* = 90 results per active substance/metabolite. Active substances and metabolites were divided into three categories based on their DT_50_. More precisely, the persistence was considered to be low if the DT_50_ was less than 30 days, medium if the DT_50_ was between 30 and 100 days and high if the DT_50_ was more than 100 days. A 4th category was included for the AS and metabolites whose DT_50_ values were not found in literature

As shown in Fig. 5, the proportion of cases where the AS or metabolite was applied and detected was relatively low. The only compounds where the proportion of cases were higher than 25% were acetamiprid (50.0%), acetamiprid-N-desmethyl (48.9%), AMPA (68.9%), difenoconazole (58.9%), flonicamid (27.8%), glyphosate (67.8%) and pyriproxyfen (25.6%). This result is not surprising as glyphosate is one of the most commonly used herbicides, and both conazole fungicides (like difenoconazole, penconazole and tebuconazole) and neonicotinoid insecticides (like acetamiprid) are often used (Humann-Guilleminot et al., 2019; Šudoma et al., 2021; Zhang et al., 2023). A high persistence in soil would also help to explain these results.

The class referring to applied but not detected showed a low proportion of cases. Flonicamid and abamectin were the substances with the highest proportions of cases, 20.0% and 11.1%, respectively. As these AS have a low DT_50_, which is 1.8 days for both (EFSA, 2023), the decay between application and sampling may help to explain the proportions of cases. Other dissipation processes, such as leaching, could also help to explain this result.

Fluopyram, pendimethalin, tebuconazole and acetamiprid were the substances with the highest proportion of cases where, despite their application not having been reported, they were still detected. Applications from previous years, drift or runoff from neighbouring orchards are possible explanations. In fact, 30 to 50% of the pesticide aerosols may not reach their target, being lost to the soil, to neighbouring fields or volatilised into the atmosphere (Eugenio et al., 2018; Riedo et al., 2021). The atmospheric deposition and the prevalence in soil from applications done in previous years are other complementary or possible explanations (Kosubová et al., 2020; Riedo et al., 2022, 2023). The last one may be especially relevant for fluopyram, pendimethalin and tebuconazole because of their high DT_50_ values which are 309 days (University of Hertfordshire, 2023), 187 days and 91.6 days, respectively (EFSA, 2023).

The ‘neither applied nor detected’ class of compounds was found in the highest proportions for most of the substances. Exceptions to this trend were acetamiprid (1.1%), acetamiprid-N-desmethyl (21.1%), AMPA (0%), difenoconazole (2.2%), fluopyram (0%) and glyphosate (0%). It should be noted that all the results from pirimicarb and pirimicarb-desmethyl are included into this category.

### Comparison between predicted and measured environmental concentrations

Both PECs and MECs of cyprodinil, deltamethrin, fenpyroximate, fluopyram and spinetoram were below the LOQ in all samples (Figure S2a, Supplementary Material). Regarding AMPA, chlorantraniliprole, difenoconazole, glyphosate, myclobutanil, pendimethalin and tebuconazole, both PECs and MECs were equal than or above the LOQ in all samples. The category where PECs were below and MECs were equal to or above LOQ was predominant for 2,4-D, acetamiprid, dodine, MCPA and triflumuron. The category where PECs were equal to or above and MECs were below the LOQ included all cases, or the majority of them, for cyprodinil, deltamethrin, fenpyroximate, fluopyram and spinetoram. In most orchards (Figure S2b, Supplementary Material), the category where PECs and MECs were equal to or above LOQ included the majority of cases.

**Fig. 6:**
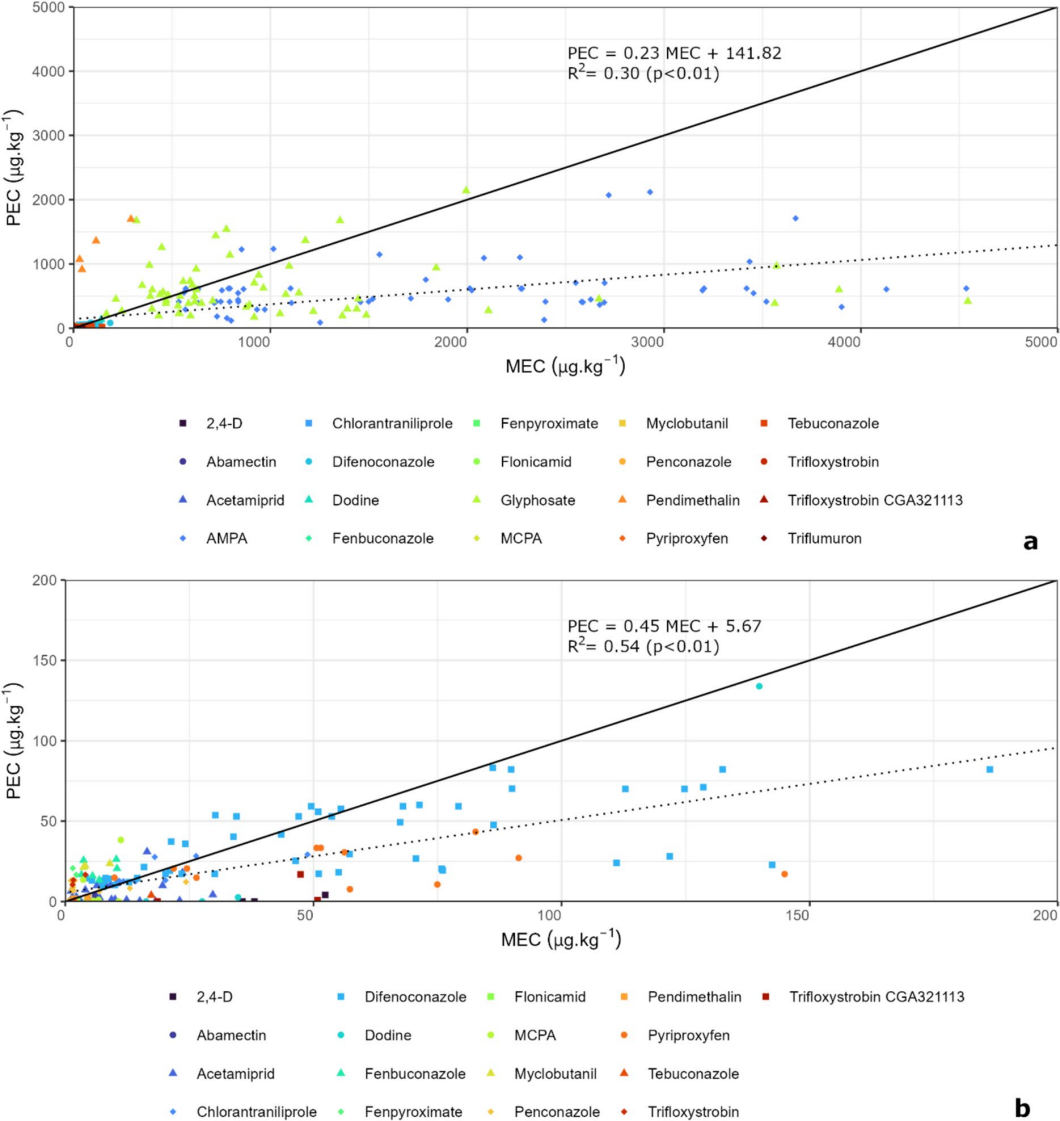
Comparison between predicted and measured environmental concentrations (PEC and MEC), with all active substances and metabolites (**a**) and excluding glyphosate, aminomethylphosphonic acid (AMPA) and pendimethalin (**b**). The solid line represents the cases where PEC = MEC. The dotted line, its equation and its coefficient of determination represent the results of the linear regression

The comparison between the predicted and the measured environmental concentrations (PECs and MECs) indicated that the decay of AS and metabolites was slower than expected, as shown by the low value of the slope in Fig. 6a and b. Glyphosate and its main metabolite, AMPA, stand out as examples of this, especially due to their high MECs. The MEC values for pendimethalin were generally lower than predicted, indicating that its decay was faster than expected. Even if glyphosate, AMPA and pendimethalin were excluded from the plot and the axis zoomed in, the general tendency of a slower than expected decay can still be observed (Fig. 6b). In this case, difenoconazole, tebuconazole and abamectin stand out as other examples of AS with relatively high values of PEC and MEC whose decay is slower than predicted. Despite of this, in more than 50% of cases, the PECs of difenoconazole were below 125% of the corresponding MECs (Figure S3a, Supplementary Material).

The analysis of Figure S3a (Supplementary Material) indicate that the most extreme cases where PEC was below MEC, i.e. the cases where PEC was below 10% of MEC, occurred mainly in 2,4-D, acetamiprid, dodine, flonicamid and triflumuron. In addition to this, in more than half of the analysed AS and metabolites, PEC was below 75% of MEC. Regarding fenbuconazole, fenpyroximate, myclobutanil, penconazole, pendimethalin and trifloxystrobin-CGA321113, in more than half of the cases, their PECs were above 125% of MECs. The comparison between PECs and MECs in the sampled orchards (Figure S3b, Supplementary Material) indicated that, in most cases, PECs were below 75% of MECs. The category that included the higher proportion of cases was that where PECs were between 10 and 50% of MECs.

As shown above, PECs were generally lower than MECs, which was similar to what was found in other studies (Beriot et al., 2023; Froger et al., 2023; Riedo et al., 2021). At this regard, Beriot et al. (2023), in Spanish samples collected in vegetable farms (0–10 cm and 10–30 cm depth), indicated that the measured pesticide concentrations in soil were generally higher than what was predicted considering the pesticides that had been applied. In the same line, Froger et al. (2023), in French soils under multiple land uses (0–20 cm depth), also reported concentrations higher than expect for, among others, difenoconazole, fluopyram and glyphosate. Adding to this, Riedo et al. (2021) found between 3 and 16 different pesticide residues in soil samples (0–5 cm depth) collected in Swiss fields under organic farming for more than 20 years.

Two main reasons may explain our results. (1) Additional sources of the substance may have been present. These potential sources may have occurred between the recorded application and the sampling time which could compensate for its decay. Drift to and from neighbouring plots, dry/wet deposition and runoff could be examples of these additional sources. (2) The particular biological, soil and climatic conditions of this region may have contributed to a different, often slower, decay. In fact, both physicochemical characteristics of pesticides and soil influence their decay. For example, high clay content and low soil pH and moisture are unfavourable for pesticide decomposition because these soils tend to show a lower microbial activity and a higher capacity to sorb pesticide substances (Beriot et al., 2023; Ismail et al., 2012; Riedo et al., 2021). Although the soils in this region do not have a particularly high clay content, they are generally acidic, and the summers are typically warm and dry which is not favourable for microbial activity and, therefore, for pesticide decay (Beriot et al., 2023; Ismail et al., 2012; Riedo et al., 2021).

### Residue background in soil

**Fig. 7:**
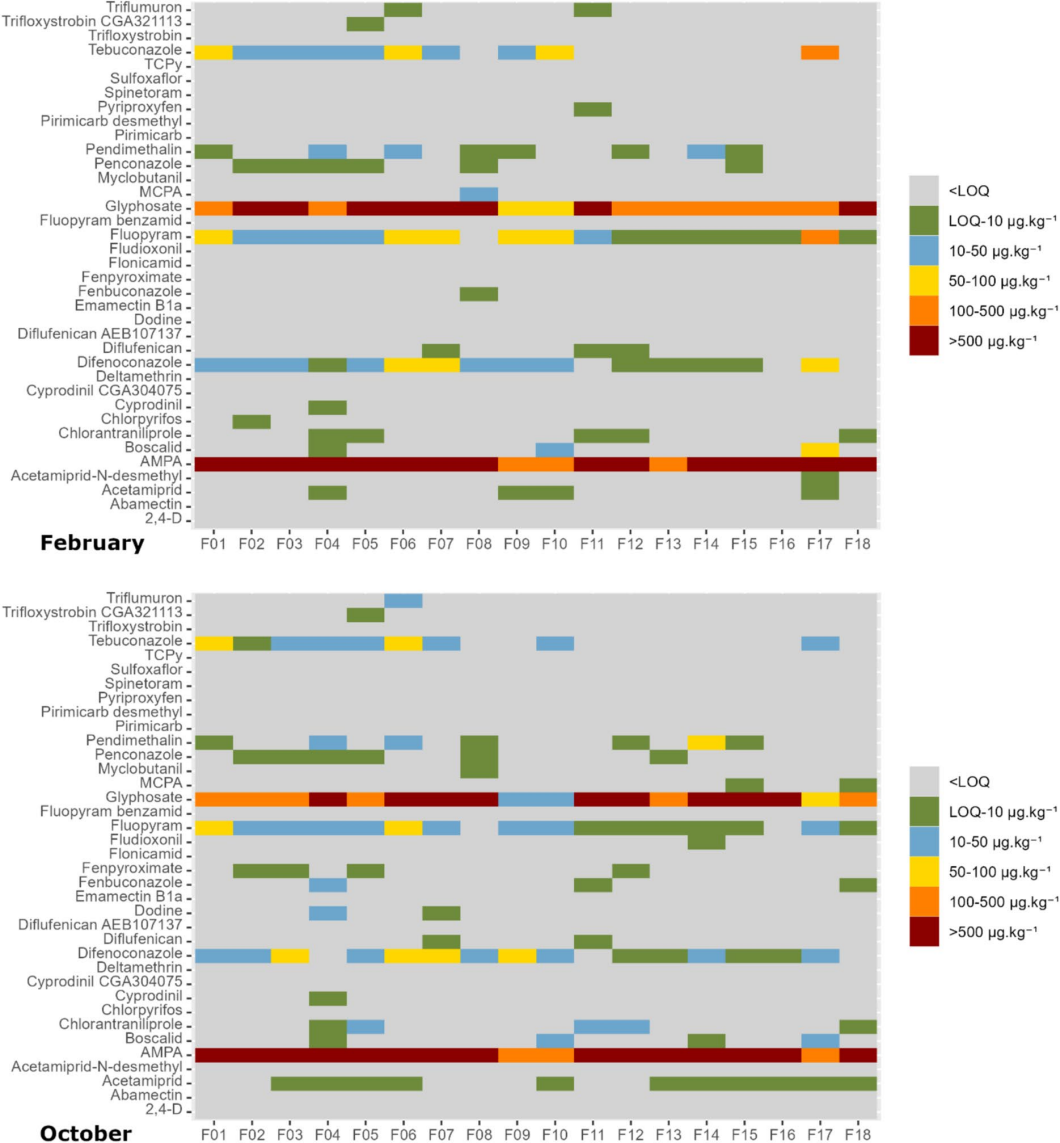
Concentrations of active substances and metabolites in the soil samples collected in February (top) and October 2022 (bottom) in each one of the 18 studied peach orchards located in the east-central Portuguese region of Beira Interior. The rows represent the active substances and metabolites, and the columns represent the orchards. The concentrations were divided into six categories, < LOQ, LOQ-10 µg.kg^−1^, 10–50 µg.kg^-1^, 50–100 µg.kg^−1^, 100–500 µg.kg^−1^ and > 500 µg.kg.^−1^. The limit of quantification (LOQ) is variable among the active substances and metabolites that were analysed and is shown in Table S1 (Supplementary material)

In the studied region, pesticide applications in peach orchards usually occur from spring to summer. As the soil samples were collected from February to October, the first samples were expected to indicate the carryover from the previous year(s) (Fig. 7). According to the application records from the studied orchards and from the list of analysed substances, only glyphosate and pyriproxyfen were applied before the collection of the first samples. Additionally, as shown in Table S3 (Supplementary Material), these AS were applied only 1 time, each one in only 1 orchard (glyphosate in F18 and pyriproxyfen in F08). However, glyphosate and its main metabolite, AMPA, were quantified in all orchards, often in concentrations higher than 500 µg.kg^−1^ in all samples and, in particular, in the samples collected in February (Fig. 7 and Figure S4 – Supplementary material). This result, especially for the samples collected in February, indicates that these compounds had been accumulating in soil over previous years. A similar result, although generally with lower concentrations, was found for difenoconazole, fluopyram and tebuconazole, whose concentrations in soil were above 10 µg.kg^−1^ in more than half of the orchards.

A pattern similar to what was described above was found in the samples collected in October (Fig. 7). In these, the soil concentrations of glyphosate and AMPA were also high in most of the orchards. There could be several possible reasons for this: (1) the AS or metabolite is highly persistent in soil, (2) the AS was applied at a high rate, and (3) the sampling was done shortly after the application of the AS.

The first point clearly applies to AMPA, whose DT_50_ is 633 days (Table S2, Supplementary material). However, the DT_50_ of glyphosate is much lower (16.11 days) (University of Hertfordshire, 2023). The results shown in Fig. 6 (and discussed in the previous section) indicate that the degradation of glyphosate under the edaphoclimatic conditions of this study is often slower than predicted.

The second point above could also contribute to the high concentrations of glyphosate and AMPA found in soil as the application rate of glyphosate (360–2160 g.ha^−1^, Fig. 2) was among the highest in the studied orchards, which was also the case in other studies (Chiaia-Hernandez et al., 2017; Geissen et al., 2021; Silva et al., 2019). If we consider the cumulative application rate, i.e. the sum of all application rates for each orchard, the interval of values varies from 450 g.ha^−1^ in F14 to 3720 g.ha^−1^ in F06 (Table S3 Supplementary material). However, the concentrations of glyphosate and AMPA were higher than 500 µg.kg^−1^ for all sampling times in both orchards (Fig. 6 and Figure S4, Supplementary material), which indicates that this point alone does not thoroughly explain the high concentrations found for glyphosate and AMPA.

The third point can be discarded, especially in what concerns the results from the first (February) and the last (October) samples. As mentioned above, the application of glyphosate before the first sampling occurred only in one orchard (F18). Moreover, the latest recorded application of glyphosate was on the 26th of May (in orchard F05), and, for most of the orchards, this herbicide was applied much sooner, in the end of March (Table S3, Supplementary material).

Concerning what was discussed above, high persistence in soil and/or high application rates could explain the fact that both glyphosate and its main metabolite (AMPA) were measured in soil often in high concentrations. The high concentrations found for all sampling times both in orchards with the lowest and the highest application rates suggest that the application rate alone does not explain the high concentrations of these two compounds found in soil. In any case, the relative importance of each one of these two points remains unclear and should be addressed in future studies.

### Limitations, remarks and suggestions for future research

The pesticide compounds analysed in this study included most of the AS reported to have been applied in 2022, in the monitored orchards along with a few metabolites. It included some AS and metabolites that were not reported to have been applied in that year but that were relevant for the assessment considering their persistency. However, the list of analysed compounds is necessarily incomplete because not only it does not include all the AS that were applied, but it also may not include some compounds that may have been applied in neighbouring plots or in previous years. Future surveys should consider (1) a higher number of case study sites, including other crops; (2) a higher number of AS, including a higher number of banned compounds, some known to be highly persistent; and (3) a more comprehensive list of metabolites. In particular, they should include compounds whose potential for accumulation in soil has been identified by several studies. This is the case, for example, of glyphosate, AMPA, conazoles and fluopyram (e.g. Beriot et al., 2023; Froger et al., 2023; Kosubová et al., 2020; Silva et al., 2019).

The inclusion of five sampling times spread throughout a whole growing season which included nearly all pesticide applications is uncommon among similar studies but, together with the pesticide application records, proved essential to better characterise pesticide decay in soil. This knowledge relates to several EU policies, such as the EU Soil Strategy for monitoring soil health (European Commission, 2023b). Our work can be useful as a case study whose results can be applicable in other regions, especially those with similar edaphoclimatic conditions. Supporting this is the fact that most of the AS are neither crop nor region/country specific, instead being used in several different crops and in several different regions and countries (Geissen et al., 2021; Silva et al., 2019; Tona et al., 2018).

Our results suggested that, for this region, the predicted concentrations of approximately 70% of the studied pesticide compounds were underestimated. Discrepancies between measured and predicted concentrations of pesticide compounds are not surprising if we consider that their decay is often carried out by the soil microbiome which is highly dependent on local factors such as temperature, humidity and soil texture. As the expected decay of an AS is an important factor for its approval in the EU, this suggests that the evaluation and approval of an AS should consider its local variations. Moreover, the general absence of quality standards for soil in EU legislation, particularly concerning pesticide residues, was already pointed out by other studies (Geissen et al., 2021; Silva et al., 2019) and hinders the appraisal of pesticide concentrations in light of their potential risks.

The kinetic parameters used in our study to determine the predicted concentrations of pesticide compounds (Table S2, Suplementary Material) were taken, mostly, from the EFSA reports (EFSA, 2023) and, for most compounds, corresponded to first-order kinetics. However, other models might result in more accurate predictions regarding pesticide decay. A comprehensive study of the decay of several pesticide compounds under the specific conditions of this region and the development of accurate models that describe and predict the decay are recommended.

## Conclusions

This study focused on peach orchards in east-central Portugal and studied the contents of pesticide residues in soil based on the farmers’ application records.

Globally, the decay of pesticide compounds was found to be slower than what was expected by the kinetic models that were used. Therefore, it can be concluded that those models, which are used by EFSA for the approval of AS, underestimate the pesticide concentrations in the soil from this region. This result highlights the need to consider local/regional variations in edaphoclimatic conditions when predicting the concentrations of AS and their metabolites. This is especially relevant if we consider that these predictions are used during the process of AS approval.

Some AS and metabolites, like glyphosate, AMPA and fluopyram, were found in all samples and, therefore, in all studied orchards, even in those where they were not recorded to have been applied in 2022. This result suggests that the importance of diffuse sources should not be underestimated. Furthermore, the monitoring of pesticide content in soil should be considered not only in agricultural fields where these substances are applied regularly, but also in other fields, which may be recipients of indirect pesticide contamination.

The results from our study also show a high potential for persistence of some AS and metabolites, namely glyphosate, AMPA, difenoconazole, tebuconazole and fluopyram, even considering that some of the AS have relatively low half-life values. This supports what was already mentioned regarding the generally slower than predicted decay of AS and metabolites in the studied region, i.e. that the decay rate of AS and metabolites cannot be accurately generalised. Instead, it should be considered on a finer scale, as it depends on the climate and soil conditions of each region.

## Supplementary Information

Below is the link to the electronic supplementary material.

Supplementary file1 (DOCX 874 KB)

## Data Availability

Data will be made available on request.
