# Supplementary material for: Dynamics of pesticide residues in soils during the growing season: a case study in peach orchards, east-central Portugal
Source: Environ Monit Assess. 2025 Feb 13;197(3):285. doi: 10.1007/s10661-025-13698-z (PMC11825562; doi:10.1007/s10661-025-13698-z)
Supplement: Supplementary file 1 — Supplementary file1 (DOCX 874 KB) [file 10661_2025_13698_MOESM1_ESM.docx]

**Pesticide residues in soils – Monitoring of levels during the growing season – A case study in peach orchards, central-east Portugal**

Abel Veloso^1,2,3*^, Vera Silva^1^, Rima Osman^1^, Maria Paula Simões^2,3^, Maria do Carmo Horta^2,3^, and Violette Geissen^1^

^1^Soil Physics and Land Management Group, Wageningen University and Research, Wageningen, the Netherlands

^2^Polytechnic Institute of Castelo Branco, School of Agriculture, Castelo Branco, Portugal

^3^Research Centre for Natural Resources, Environment and Society (CERNAS), Castelo Branco, Portugal

*Corresponding author ([abel.veloso@wur.nl](mailto:abel.veloso@wur.nl))

# **Supplementary material**

| 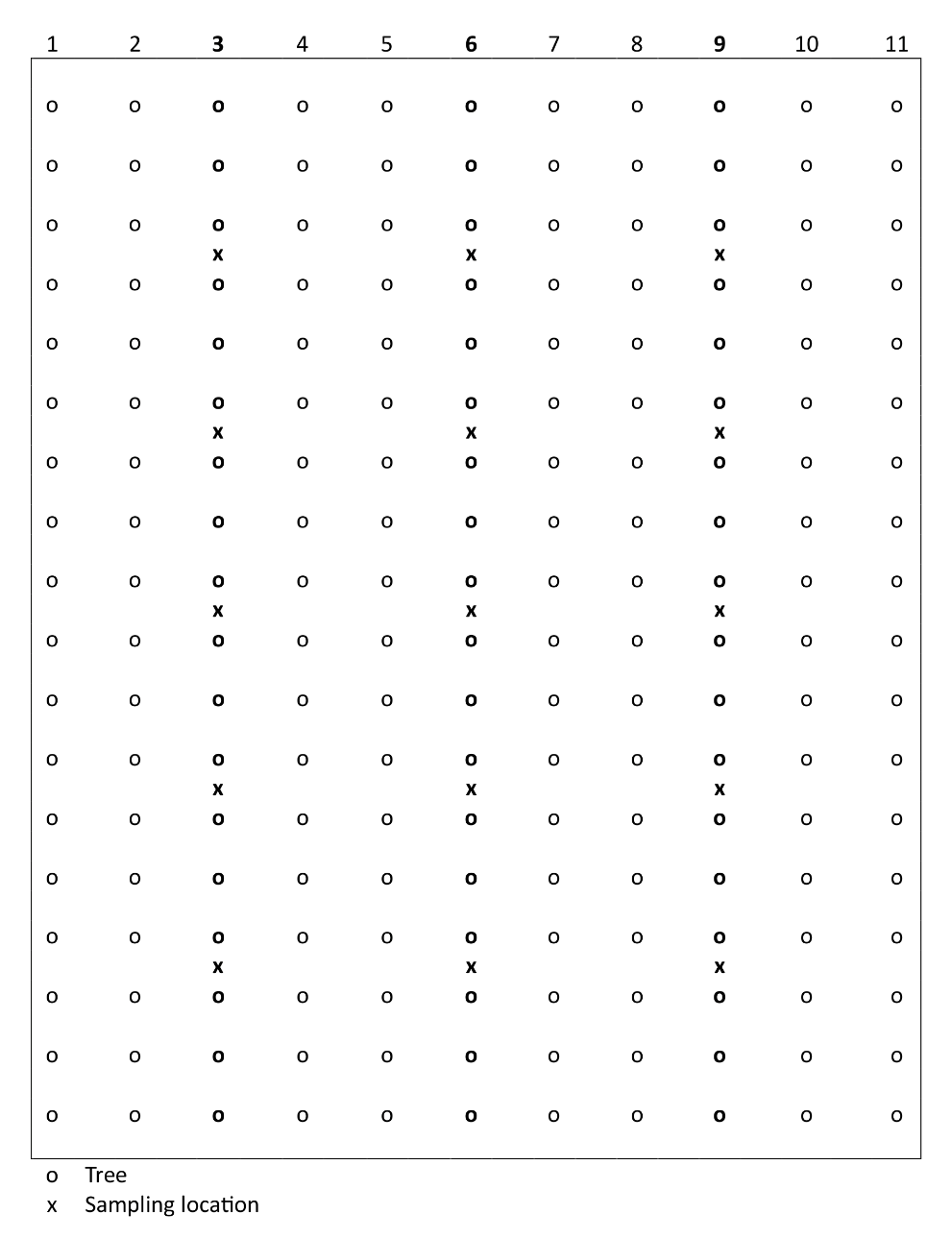 |
| --- |
| **Fig. S1** Schematic represention of the sample locations within the orchards located in the east-central Portuguese region of Beira Interior. To maximize the representativeness of the sampling, each orchard was divided in 4 parts. The 3 composite samples were collected in the 3 inner rows (represented here in bold). Each composite sample was formed by mixing 5 sub-samples collected in 2022, at even intervals within each sampling row. |

**Table S1** Limits of detection (LOD) and quantification (LOQ), median, maximum and frequencies (absolute and relative) for each pesticide residue that was analysed. These results were compiled from the average of the pesticide content obtained for the 3 composite samples collected per orchard and per month and are listed in alphabetic order according to the name of the pesticide residue. Only contents higher than LOQ were considered for the determination of median and maximum values. The soil samples were collected in 2022, in each one of the 18 studied peach orchards located in the east-central Portuguese region of Beira Interior.

| Pesticide residue | LOD  (µg.kg^-1^) | LOQ  (µg.kg^-1^) | Median  (µg.kg^-1^) | Maximum  (µg.kg^-1^) | Number of results ≥LOD  (% of 90 samples) | Number of results ≥LOQ  (% of 90 total) |
| --- | --- | --- | --- | --- | --- | --- |
| 2,4-D | 1.4 | 5.0 | 43.5 | 369.1 | 13 (14.4%) | 8 (8.9%) |
| Abamectin | 1.4 | 5.0 | <LOQ | <LOQ | 1 (1.1%) | 0 (0%) |
| Acetamiprid | 0.1 | 1.0 | 4.6 | 29.7 | 89 (98.9%) | 55 (61.1%) |
| Acetamiprid-N-desmethyl | 0.1 | 1.0 | 1.1 | 3.0 | 71 (78.9%) | 14 (15.6%) |
| AMPA | 10.0 | 25.0 | 1058.9 | 4649.3 | 90 (100%) | 90 (100%) |
| Boscalid | 0.4 | 1.0 | 18.2 | 72.4 | 30 (33.3%) | 17 (18.9%) |
| Chlorantraniliprole | 0.2 | 1.0 | 6.3 | 48.8 | 44 (48.9%) | 25 (27.8%) |
| Chlorpyrifos | 0.9 | 2.5 | 3.6 | 7.2 | 10 (11.1%) | 5 (5.6%) |
| Cyprodinil | 0.9 | 1.0 | 2.1 | 4.2 | 5 (5.6%) | 5 (5.6%) |
| Cyprodinil-CGA304075 | 0.3 | 1.0 | <LOQ | <LOQ | 0 (0%) | 0 (0%) |
| Deltamethrin | 0.6 | 2.5 | <LOQ | <LOQ | 1 (1.1%) | 0 (0%) |
| Difenoconazole | 0.1 | 1.0 | 35.7 | 186.3 | 88 (97.8%) | 80 (88.9%) |
| Diflufenican | 0.2 | 1.0 | 1.6 | 3.6 | 30 (33.3%) | 12 (13.3%) |
| Diflufenican-AEB107137 | 1.5 | 5.0 | <LOQ | <LOQ | 2 (2.2%) | 0 (0%) |
| Dodine | 4.0 | 5.0 | 17.3 | 139.8 | 9 (10%) | 8 (8.9%) |
| Emamectin-B1a | 0.9 | 1.0 | <LOQ | <LOQ | 0 (0%) | 0 (0%) |
| Fenbuconazole | 1.5 | 2.5 | 8.6 | 38.8 | 13 (14.4%) | 12 (13.3%) |
| Fenpyroximate | 0.1 | 1.0 | 3.2 | 5.6 | 18 (20%) | 8 (8.9%) |
| Flonicamid | 0.1 | 1.0 | 4.3 | 9.2 | 32 (35.6%) | 7 (7.8%) |
| Fludioxonil | 0.1 | 1.0 | 3.6 | 3.6 | 6 (6.7%) | 1 (1.1%) |
| Fluopyram | 0.2 | 1.0 | 29.8 | 165.2 | 90 (100%) | 84 (93.3%) |
| Fluopyram-benzamide | 0.1 | 1.0 | 2.3 | 5.2 | 43 (47.8%) | 4 (4.4%) |
| Glyphosate | 10.0 | 25.0 | 584.1 | 4542.6 | 90 (100%) | 90 (100%) |
| MCPA | 0.7 | 2.5 | 8.0 | 401.4 | 23 (25.6%) | 17 (18.9%) |
| Myclobutanil | 0.6 | 1.0 | 4.0 | 9.0 | 3 (3.3%) | 3 (3.3%) |
| Penconazole | 0.8 | 1.0 | 3.2 | 24.3 | 34 (37.8%) | 32 (35.6%) |
| Pendimethalin | 0.3 | 1.0 | 4.7 | 291.6 | 56 (62.2%) | 36 (40%) |
| Pirimicarb | 0.0 | 1.0 | <LOQ | <LOQ | 0 (0%) | 0 (0%) |
| Pirimicarb-desmethyl | 0.0 | 1.0 | <LOQ | <LOQ | 0 (0%) | 0 (0%) |
| Pyriproxyfen | 0.1 | 1.0 | 1.3 | 1.8 | 31 (34.4%) | 4 (4.4%) |
| Spinetoram | 1.7 | 5.0 | <LOQ | <LOQ | 0 (0%) | 0 (0%) |
| Sulfoxaflor | 0.0 | 1.0 | <LOQ | <LOQ | 13 (14.4%) | 0 (0%) |
| TCPy | 0.5 | 5.0 | <LOQ | <LOQ | 12 (13.3%) | 0 (0%) |
| Tebuconazole | 1.4 | 5.0 | 24.5 | 144.9 | 60 (66.7%) | 49 (54.4%) |
| Trifloxystrobin | 0.0 | 1.0 | 9.4 | 17.3 | 9 (10%) | 2 (2.2%) |
| Trifloxystrobin-CGA321113 | 0.3 | 1.0 | 1.3 | 4.0 | 5 (5.6%) | 4 (4.4%) |
| Triflumuron | 0.2 | 1.0 | 7.2 | 50.8 | 11 (12.2%) | 7 (7.8%) |

**Table S2** Kinetic parameters used for the determination of predicted environmental concentrations (PEC). Two sources were considered for kinetic parameters. The primary source was the EFSA reports. When data was not found here, the kinetic parameters were obtained from the Pesticide Properties Database/PPDB (University of Hertfordshire, 2023).

| Pesticide residue | Kinetics | DT_50_ (days) | DT_50(1)_ (days) | DT_50(2)_ (days) | g | Source |
| --- | --- | --- | --- | --- | --- | --- |
| 2,4-D | SFO | 7.5 | n.a. | n.a. | n.a. | (EFSA, 2014) |
| Abamectin | SFO | 1.8 | n.a. | n.a. | n.a. | (EFSA, 2008a) |
| Acetamiprid | SFO | 12.96 | n.a. | n.a. | n.a. | (EFSA, 2016a) |
| AMPA | SFO | 633 | n.a. | n.a. | n.a. | (EFSA, 2015) |
| Chlorantraniliprole | SFO | 1378 | n.a. | n.a. | n.a. | (EFSA, 2013) |
| Difenoconazole | SFO | 265 | n.a. | n.a. | n.a. | (EFSA, 2011a) |
| Dodine | SFO | 10.54 | n.a. | n.a. | n.a. | (EFSA, 2010a) |
| Fenbuconazole | SFO | 172 | n.a. | n.a. | n.a. | (EFSA, 2010b) |
| Fenpyroximate | SFO | 135.1 | n.a. | n.a. | n.a. | (University of Hertfordshire, 2023) |
| Flonicamid | SFO | 1.8 | n.a. | n.a. | n.a. | (EFSA, 2010c) |
| Glyphosate | DFOP | n.a. | 18.05 | 187.34 | 0.575 | (EFSA, 2015) |
| MCPA | SFO | 12.07 | n.a. | n.a. | n.a. | (University of Hertfordshire, 2023) |
| Myclobutanil | SFO | 711.5 | n.a. | n.a. | n.a. | (EFSA, 2010d) |
| Penconazole | SFO | 115 | n.a. | n.a. | n.a. | (EFSA, 2008b) |
| Pendimethalin | SFO | 187 | n.a. | n.a. | n.a. | (EFSA, 2016b) |
| Pyriproxyfen | SFO | 25 | n.a. | n.a. | n.a. | (EFSA, 2009) |
| Tebuconazole | SFO | 91.6 | n.a. | n.a. | n.a. | (EFSA, 2008c) |
| Trifloxystrobin | SFO | 0.34 | n.a. | n.a. | n.a. | (University of Hertfordshire, 2023) |
| Trifloxystrobin-CGA321113 | SFO | 122.4 | n.a. | n.a. | n.a. | (University of Hertfordshire, 2023) |
| Triflumuron | SFO | 14.6 | n.a. | n.a. | n.a. | (EFSA, 2011b) |

n.a. – not applicable, SFO – single first order, DFOP – double first order in parallel, DT_50_ – time required for the dissipation of 50% of the initial concentration

## **Table S3** Synthesis of the application records provided by the farmers for the sampling year (2022) for the 18 peach orchards from the east-central Portuguese region of Beira Interior. The active substances that were quantified are highlighted in grey.

| Orchard | Month of application | Day/interval of application | Active Substance | Type | Commercial product | Application rate of active substance (g.ha^-1^) |
| --- | --- | --- | --- | --- | --- | --- |
| F01 | February | 10-16 | Copper salts | Fungicide | Cuprocol | 2800 |
| F01 | February | 23-24 | Ziram | Fungicide | Zidora AG | 1520 |
| F01 | March | 13-14 | Cyprodinil | Fungicide | Chorus 50 WG | 250 |
| F01 | March | 26-30 | Glyphosate | Herbicide | Roundup Flex | 1920 |
| F01 | March | 29-30 | Ziram | Fungicide | Zidora AG | 1520 |
| F01 | April | 14-15 | Flonicamid | Insecticide/ acaricide | Teppeki | 70 |
| F01 | April | 14-15 | Sulphur | Fungicide | Azufega 80 LA | 2400 |
| F01 | May | 2 | Sulphur | Fungicide | Azufega 80 LA | 2400 |
| F01 | May | 24-25 | Sulfoxaflor | Insecticide/ acaricide | Closer | 36 |
| F01 | June | 11-12 | Spinetoram | Insecticide/ acaricide | Delegate 250 WG | 75 |
| F01 | July | 17 | Lambda-cyalothrin | Insecticide/ acaricide | Karate zeon | 12,5 |
| F01 | July | 17 | Tebuconazole | Fungicide | Flint Max | 150 |
| F01 | July | 17 | Trifloxystrobin | Fungicide | Flint Max | 75 |
| F02 | February | 20 | Copper salts | Fungicide | Cuprocol | 3500 |
| F02 | March | 1 | Ziram | Fungicide | Zidora AG | 1520 |
| F02 | March | 16 | Acetamiprid | Insecticide/ acaricide | Carnadine | 70 |
| F02 | March | 16 | Ziram | Fungicide | Zidora AG | 1520 |
| F02 | March | 16 | Difenoconazole | Fungicide | Score 250 EC | 75 |
| F02 | March | 25 | Difenoconazole | Fungicide | Score 250 EC | 75 |
| F02 | April | 7 | Ziram | Fungicide | Zidora AG | 1520 |
| F02 | April | 14 | Sulfoxaflor | Insecticide/ acaricide | Closer | 36 |
| F02 | April | 14 | Difenoconazole | Fungicide | Zanol | 75 |
| F02 | April | 22 | Glyphosate | Herbicide | Touchdown Premium | 1440 |
| F02 | April | 28 | Sulphur | Fungicide | Thiovit Jet | 3200 |
| F02 | May | 8 | Sulphur | Fungicide | Thiovit Jet | 3200 |
| F02 | June | 3 | Fluopyram | Fungicide | Luna experience | 100 |
| F02 | June | 3 | Tebuconazole | Fungicide | Luna experience | 100 |
| F02 | June | 9 | Acetamiprid | Insecticide/ acaricide | Carnadine | 70 |
| F02 | July | 3 | Abamectin | Insecticide/ acaricide | Vertimec | 13,5 |
| F03 | February | 20 | Copper salts | Fungicide | Cuprocol | 3500 |
| F03 | March | 1 | Ziram | Fungicide | Zidora AG | 1520 |
| F03 | March | 16 | Acetamiprid | Insecticide/ acaricide | Carnadine | 70 |
| F03 | March | 16 | Ziram | Fungicide | Zidora AG | 1520 |
| F03 | March | 16 | Difenoconazole | Fungicide | Score 250 EC | 75 |
| F03 | March | 22 | Glyphosate | Herbicide | Touchdown Premium | 1440 |

## **Table S3 (cont.)** Synthesis of the application records provided by the farmers for the sampling year (2022) for the 18 peach orchards from the east-central Portuguese region of Beira Interior. The active substances that were quantified are highlighted in grey.

| F03 | March | 25 | Difenoconazole | Fungicide | Score 250 EC | 75 |
| --- | --- | --- | --- | --- | --- | --- |
| F03 | April | 7 | Ziram | Fungicide | Zidora AG | 1520 |
| F03 | April | 14 | Flonicamid | Insecticide/ acaricide | Teppeki | 70 |
| F03 | April | 14 | Difenoconazole | Fungicide | Zanol | 75 |
| F03 | April | 28 | Sulphur | Fungicide | Thiovit Jet | 3200 |
| F03 | May | 8 | Sulphur | Fungicide | Thiovit Jet | 3200 |
| F03 | June | 3 | Acetamiprid | Insecticide/ acaricide | Starpride Plus | 100 |
| F03 | June | 3 | Fluopyram | Fungicide | Luna experience | 100 |
| F03 | June | 3 | Tebuconazole | Fungicide | Luna experience | 100 |
| F04 | February | 25 | Paraffin oil | Insecticide/ acaricide | Garbol | 7900 |
| F04 | February | 25 | Copper salts | Fungicide | Hidrotec 20% HI BIO | 1000 |
| F04 | March | 26 | Deltamethrin | Insecticide/ acaricide | Deltagronis | 12,5 |
| F04 | March | 26 | Ziram | Fungicide | Zidora AG | 1520 |
| F04 | April | 13 | Dodine | Fungicide | Syllit 544 SC | 897,6 |
| F04 | April | 18 | Pendimethalin | Herbicide | Pendinova | 1320 |
| F04 | April | 18 | Glyphosate | Herbicide | Clinic Direct 360 | 1440 |
| F04 | May | 8 | Deltamethrin | Insecticide/ acaricide | Deltagronis | 12,5 |
| F04 | May | 8 | Sulphur | Fungicide | Thiovit Jet | 3200 |
| F04 | May | 15 | Sulphur | Fungicide | Thiovit Jet | 3200 |
| F04 | June | 1 | Acetamiprid | Insecticide/ acaricide | Carnadine | 100 |
| F04 | June | 15 | Acetamiprid | Insecticide/ acaricide | Epik SL | 100 |
| F04 | July | 2 | Deltamethrin | Insecticide/ acaricide | Decis Evo | 12,5 |
| F05 | January | 6 | Copper salts | Fungicide | Vitra 40 | 2000 |
| F05 | February | 18 | Paraffin oil | Insecticide/ acaricide | Sensei | 8170 |
| F05 | February | 18 | Copper salts | Fungicide | Vitra 40 | 1600 |
| F05 | February | 18 | Pyriproxyfen | Insecticide/ acaricide | Admiral | 50 |
| F05 | March | 2 | Ziram | Fungicide | Zidora AG | 1786 |
| F05 | March | 11 | Ziram | Fungicide | Zidora AG | 1786 |
| F05 | March | 17 | Flonicamid | Insecticide/ acaricide | Teppeki | 70 |
| F05 | March | 30 | Glyphosate | Herbicide | Touchdown Premium | 360 |
| F05 | April | 7 | Difenoconazole | Fungicide | Score 250 EC | 75 |
| F05 | April | 19 | Difenoconazole | Fungicide | Score 250 EC | 75 |
| F05 | April | 29 | Sulphur | Fungicide | Thiovit Jet | 3200 |
| F05 | May | 13 | Penconazole | Fungicide | Topaze | 40 |
| F05 | May | 13 | Acetamiprid | Insecticide/ acaricide | Epik SL | 100 |
| F05 | May | 25 | Sulphur | Fungicide | Thiovit Jet | 3200 |

## **Table S3 (cont.)** Synthesis of the application records provided by the farmers for the sampling year (2022) for the 18 peach orchards from the east-central Portuguese region of Beira Interior. The active substances that were quantified are highlighted in grey.

| F05 | May | 25 | Chlorantraniliprole | Insecticide/ acaricide | Voliam | 40 |
| --- | --- | --- | --- | --- | --- | --- |
| F05 | May | 26 | Glyphosate | Herbicide | Touchdown Premium | 360 |
| F05 | June | 12 | Acetamiprid | Insecticide/ acaricide | Carnadine | 80 |
| F05 | June | 26 | Trifloxystrobin | Fungicide | Flint | 75 |
| F05 | June | 26 | Abamectin | Insecticide/ acaricide | Voliam Targo | 13,5 |
| F05 | June | 26 | Chlorantraniliprole | Insecticide/ acaricide | Voliam Targo | 33,75 |
| F05 | July | 20 | Fenpyroximate | Insecticide/ acaricide | Dinamite | 64 |
| F05 | July | 20 | Abamectin | Insecticide/ acaricide | Vertimec | 13,5 |
| F05 | July | 30 | Lambda-cyalothrin | Insecticide/ acaricide | Karate zeon | 12,5 |
| F05 | November | 4 | Copper salts | Fungicide | Vitra 40 | 2000 |
| F05 | November | 18 | Copper salts | Fungicide | Vitra 40 | 2000 |
| F05 | November | 28 | Copper salts | Fungicide | Vitra 40 | 2000 |
| F06 | February | 13-22 | Copper salts | Fungicide | Kocide 35 DF | 1225 |
| F06 | March | 5-12 | Glyphosate | Herbicide | Clinic Direct 360 | 360 |
| F06 | March | 5-12 | 2,4-D | Herbicide | Kyleo | 800 |
| F06 | March | 5-12 | Glyphosate | Herbicide | Kyleo | 1200 |
| F06 | March | 7 | Ziram | Fungicide | Zidora AG | 1520 |
| F06 | March | 16 | Tau-fluvalinate | Insecticide/ acaricide | Klartan | 80 |
| F06 | March | 16 | Difenoconazole | Fungicide | Zanol | 75 |
| F06 | March | 25 | Ziram | Fungicide | Zidora AG | 1520 |
| F06 | April | 3-6 | Flonicamid | Insecticide/ acaricide | Teppeki | 70 |
| F06 | April | 3-6 | Fluopyram | Fungicide | Luna experience | 80 |
| F06 | April | 3-6 | Tebuconazole | Fungicide | Luna experience | 80 |
| F06 | April | 28 | Sulphur | Fungicide | Stulln WG Advance | 3200 |
| F06 | May | 17 | Acetamiprid | Insecticide/ acaricide | Carnadine | 100 |
| F06 | May | 17 | Sulphur | Fungicide | Stulln WG Advance | 3200 |
| F06 | May | 20-25 | Glyphosate | Herbicide | Roundup Ultramax | 2160 |
| F06 | June | 6 | Triflumuron | Insecticide/ acaricide | Alsystin Max | 120 |
| F06 | June | 6 | Sulphur | Fungicide | Stulln WG Advance | 3200 |
| F06 | August | 12 | Lambda-cyalothrin | Insecticide/ acaricide | Judo | 12,5 |
| F07 | February | 20-23 | Glyphosate | Herbicide | Clinic Direct 360 | 1440 |
| F07 | February | 23 | Copper salts | Fungicide | Cuprocol | 2800 |
| F07 | March | 10 | Ziram | Fungicide | Zidora AG | 1520 |
| F07 | March | 23 | Ziram | Fungicide | Zidora AG | 1520 |
| F07 | April | 4 | Flonicamid | Insecticide/ acaricide | Teppeki | 70 |

## **Table S3 (cont.)** Synthesis of the application records provided by the farmers for the sampling year (2022) for the 18 peach orchards from the east-central Portuguese region of Beira Interior. The active substances that were quantified are highlighted in grey.

| F07 | April | 4 | Difenoconazole | Fungicide | Score 250 EC | 75 |
| --- | --- | --- | --- | --- | --- | --- |
| F07 | April | 13 | Sulphur | Fungicide | Azufega | 3940 |
| F07 | May | 24 | Acetamiprid | Insecticide/ acaricide | Carnadine | 100 |
| F07 | May | 24 | Sulphur | Fungicide | Azufega | 3940 |
| F08 | February | 15-17 | Pyriproxyfen | Insecticide/ acaricide | Blade | 50 |
| F08 | February | 15-17 | Copper salts | Fungicide | Cuprocol | 3500 |
| F08 | February | 15-17 | Paraffin oil | Insecticide/ acaricide | Ovitex | 16340 |
| F08 | March | 5-8 | Ziram | Fungicide | Zidora AG | 1520 |
| F08 | April | 8-10 | Acetamiprid | Insecticide/ acaricide | Epik SG | 50 |
| F08 | April | 8-10 | Ziram | Fungicide | Zidora AG | 1520 |
| F08 | April | 10-23 | Glyphosate | Herbicide | Touchdown Premium | 1080 |
| F08 | April | 10-23 | MCPA | Herbicide | Herbinexa 50 | 1200 |
| F08 | April | 19-23 | Sulfoxaflor | Insecticide/ acaricide | Closer | 36 |
| F08 | April | 19-23 | Difenoconazole | Fungicide | Score 250 EC | 75 |
| F08 | May | 14-16 | Acetamiprid | Insecticide/ acaricide | Epik SG | 50 |
| F08 | May | 14-16 | Sulphur | Fungicide | Stulln WG Advance | 3200 |
| F08 | June | 9-12 | Emamectin | Insecticide/ acaricide | Affirm Opti | 14,25 |
| F08 | June | 9-12 | Myclobutanil | Fungicide | Systhane Ecozome | 59,85 |
| F09 | February | 10 | Ziram | Fungicide | Zidora AG | 1520 |
| F09 | February | 18 | Pyriproxyfen | Insecticide/ acaricide | Blade | 30 |
| F09 | February | 18 | Difenoconazole | Fungicide | Zanol | 75 |
| F09 | February | 24 | Flonicamid | Insecticide/ acaricide | Teppeki | 70 |
| F09 | February | 24 | Ziram | Fungicide | Zidora AG | 1520 |
| F09 | March | 11 | Flonicamid | Insecticide/ acaricide | Affinto | 70 |
| F09 | March | 11 | Ziram | Fungicide | Zidora AG | 1520 |
| F09 | March | 29 | Difenoconazole | Fungicide | Zanol | 75 |
| F09 | April | 2 | Acetamiprid | Insecticide/ acaricide | Starpride Plus | 100 |
| F09 | April | 20 | Difenoconazole | Fungicide | Zanol | 75 |
| F09 | May | 15 | Captan | Fungicide | Merpan 480 SC | 1200 |
| F10 | February | 10 | Ziram | Fungicide | Zidora AG | 1520 |
| F10 | February | 18 | Pyriproxyfen | Insecticide/ acaricide | Blade | 30 |
| F10 | February | 18 | Difenoconazole | Fungicide | Zanol | 75 |
| F10 | February | 24 | Flonicamid | Insecticide/ acaricide | Teppeki | 70 |
| F10 | February | 24 | Ziram | Fungicide | Zidora AG | 1520 |
| F10 | March | 11 | Flonicamid | Insecticide/acaricide | Affinto | 70 |

## **Table S3 (cont.)** Synthesis of the application records provided by the farmers for the sampling year (2022) for the 18 peach orchards from the east-central Portuguese region of Beira Interior. The active substances that were quantified are highlighted in grey.

| F10 | March | 11 | Ziram | Fungicide | Zidora AG | 1520 |
| --- | --- | --- | --- | --- | --- | --- |
| F10 | March | 29 | Difenoconazole | Fungicide | Zanol | 75 |
| F10 | April | 2 | Acetamiprid | Insecticide/ acaricide | Starpride Plus | 100 |
| F10 | April | 20 | Difenoconazole | Fungicide | Zanol | 75 |
| F10 | May | 15 | Captan | Fungicide | Merpan 480 SC | 1200 |
| F11 | February | 23 | Paraffin oil | Insecticide/ acaricide | Laincoil | 7900 |
| F11 | February | 23 | Pyriproxyfen | Insecticide/ acaricide | Blade | 50 |
| F11 | February | 23 | Copper salts | Fungicide | Hidrotec 20% HI BIO | 1000 |
| F11 | March | 22 | Glyphosate | Herbicide | Touchdown Premium | 1440 |
| F11 | March | 26 | Flonicamid | Insecticide/ acaricide | Affinto | 70 |
| F11 | March | 26 | Ziram | Fungicide | Zidora AG | 1520 |
| F11 | April | 14 | Ziram | Fungicide | Zidora AG | 1520 |
| F11 | May | 2 | Sulphur | Fungicide | Thiopron 825 | 3300 |
| F11 | May | 19 | Abamectin | Insecticide/ acaricide | Voliam Targo | 13,5 |
| F11 | May | 19 | Chlorantraniliprole | Insecticide/ acaricide | Voliam Targo | 33,75 |
| F11 | May | 19 | Fenbuconazole | Fungicide | Impala | 75 |
| F11 | May | 27 | Sulfoxaflor | Insecticide/ acaricide | Closer | 36 |
| F12 | February | 16 | Ziram | Fungicide | Zidora AG | 1520 |
| F12 | March | 5 | Ziram | Fungicide | Zidora AG | 1520 |
| F12 | March | 22 | Glyphosate | Herbicide | Glifotop Ultra | 1440 |
| F12 | March | 26 | Ziram | Fungicide | Zidora AG | 1520 |
| F12 | April | 5 | Difenoconazole | Fungicide | Score 250 EC | 37,5 |
| F12 | April | 30 | Sulphur | Fungicide | Kumulus S | 2400 |
| F12 | May | 28 | Abamectin | Insecticide/ acaricide | Voliam Targo | 13,5 |
| F12 | May | 28 | Chlorantraniliprole | Insecticide/ acaricide | Voliam Targo | 33,75 |
| F12 | May | 28 | Sulphur | Fungicide | Kumulus S | 2400 |
| F12 | July | 18 | Lambda-cyalothrin | Insecticide/ acaricide | Cisor | 12,5 |
| F12 | August | 17 | Lambda-cyalothrin | Insecticide/ acaricide | Karate zeon | 12,5 |
| F13 | February | 25 | Copper salts | Fungicide | Vitra 40 | 1200 |
| F13 | March | 2 | Ziram | Fungicide | Zidora AG | 1520 |
| F13 | March | 11 | Ziram | Fungicide | Zidora AG | 1520 |
| F13 | March | 18 | Ziram | Fungicide | Zidora AG | 1520 |
| F13 | March | 23 | Glyphosate | Herbicide | Montana Ascenza | 720 |
| F13 | April | 13 | Captan | Fungicide | Merpan 80 WG | 1440 |
| F13 | April | 20 | Captan | Fungicide | Merpan 80 WG | 1440 |
| F13 | May | 2 | Acetamiprid | Insecticide/ acaricide | Starpride Plus | 70 |

## **Table S3 (cont.)** Synthesis of the application records provided by the farmers for the sampling year (2022) for the 18 peach orchards from the east-central Portuguese region of Beira Interior. The active substances that were quantified are highlighted in grey.

| F13 | May | 2 | Difenoconazole | Fungicide | Cerimonia | 50 |
| --- | --- | --- | --- | --- | --- | --- |
| F13 | May | 25 | Penconazole | Fungicide | Douro 10EC | 40 |
| F14 | February | 21 | Glyphosate | Herbicide | Galaxia SL | 450 |
| F14 | February | 21 | MCPA | Herbicide | Galaxia SL | 450 |
| F14 | March | 2 | Ziram | Fungicide | Zidora AG | 1520 |
| F14 | March | 15 | Ziram | Fungicide | Zidora AG | 1520 |
| F14 | March | 25 | Difenoconazole | Fungicide | Zanol | 75 |
| F14 | April | 13 | Difenoconazole | Fungicide | Zanol | 75 |
| F14 | April | 18 | Flonicamid | Insecticide/ acaricide | Affinto | 70 |
| F15 | February | 25 | Copper salts | Fungicide | Vitra 40 | 1200 |
| F15 | March | 2 | Ziram | Fungicide | Zidora AG | 1520 |
| F15 | March | 11 | Ziram | Fungicide | Zidora AG | 1520 |
| F15 | March | 18 | Ziram | Fungicide | Zidora AG | 1520 |
| F15 | March | 23 | Glyphosate | Herbicide | Montana Ascenza | 720 |
| F15 | April | 13 | Captan | Fungicide | Merpan 80 WG | 1440 |
| F15 | April | 20 | Captan | Fungicide | Merpan 80 WG | 1440 |
| F15 | May | 2 | Acetamiprid | Insecticide/ acaricide | Starpride Plus | 70 |
| F15 | May | 2 | Difenoconazole | Fungicide | Cerimonia | 50 |
| F15 | May | 25 | Penconazole | Fungicide | Douro 10EC | 40 |
| F16 | February | 25 | Copper salts | Fungicide | Vitra 40 | 1200 |
| F16 | March | 2 | Ziram | Fungicide | Zidora AG | 1520 |
| F16 | March | 11 | Ziram | Fungicide | Zidora AG | 1520 |
| F16 | March | 18 | Ziram | Fungicide | Zidora AG | 1520 |
| F16 | March | 23 | Glyphosate | Herbicide | Montana Ascenza | 720 |
| F16 | April | 13 | Captan | Fungicide | Merpan 80 WG | 1440 |
| F16 | April | 20 | Captan | Fungicide | Merpan 80 WG | 1440 |
| F16 | May | 2 | Acetamiprid | Insecticide/ acaricide | Starpride Plus | 70 |
| F16 | May | 2 | Difenoconazole | Fungicide | Cerimonia | 50 |
| F16 | May | 25 | Penconazole | Fungicide | Douro 10EC | 40 |
| F17 | February | 10 | Ziram | Fungicide | Zidora AG | 1520 |
| F17 | February | 18 | Pyriproxyfen | Insecticide/ acaricide | Blade | 30 |
| F17 | February | 18 | Difenoconazole | Fungicide | Zanol | 75 |
| F17 | February | 24 | Flonicamid | Insecticide/ acaricide | Teppeki | 70 |
| F17 | February | 24 | Ziram | Fungicide | Zidora AG | 1520 |
| F17 | March | 11 | Flonicamid | Insecticide/ acaricide | Affinto | 70 |
| F17 | March | 11 | Ziram | Fungicide | Zidora AG | 1520 |
| F17 | March | 29 | Difenoconazole | Fungicide | Zanol | 75 |
| F17 | April | 2 | Acetamiprid | Insecticide/ acaricide | Starpride Plus | 100 |
| F17 | April | 20 | Difenoconazole | Fungicide | Zanol | 75 |
| F17 | May | 15 | Captan | Fungicide | Merpan 480 SC | 1200 |

## **Table S3 (cont.)** Synthesis of the application records provided by the farmers for the sampling year (2022) for the 18 peach orchards from the east-central Portuguese region of Beira Interior. The active substances that were quantified are highlighted in grey.

| F18 | February | 10 | Glyphosate | Herbicide | Clinic Direct 360 | 1440 |
| --- | --- | --- | --- | --- | --- | --- |
| F18 | February | 12 | Copper salts | Fungicide | Hidrotec 20% Hi Bio | 1000 |
| F18 | February | 24 | Ziram | Fungicide | Zidora AG | 1520 |
| F18 | March | 5 | Ziram | Fungicide | Zidora AG | 1520 |
| F18 | March | 16 | Ziram | Fungicide | Zidora AG | 1520 |
| F18 | March | 26 | Fenbuconazole | Fungicide | Impala | 75 |
| F18 | April | 19 | Sulphur | Fungicide | Thiopron 825 | 3300 |
| F18 | May | 9 | Flonicamid | Insecticide/ acaricide | Teppeki | 70 |
| F18 | May | 9 | Sulphur | Fungicide | Thiopron 825 | 3300 |
| F18 | May | 10 | Glyphosate | Herbicide | Halvetic | 720 |
| F18 | June | 4 | Flonicamid | Insecticide/ acaricide | Teppeki | 70 |
| F18 | June | 4 | Sulphur | Fungicide | Thiopron 825 | 3300 |
| F18 | June | 24 | Sulphur | Fungicide | Azufega 80 LA | 3200 |

| 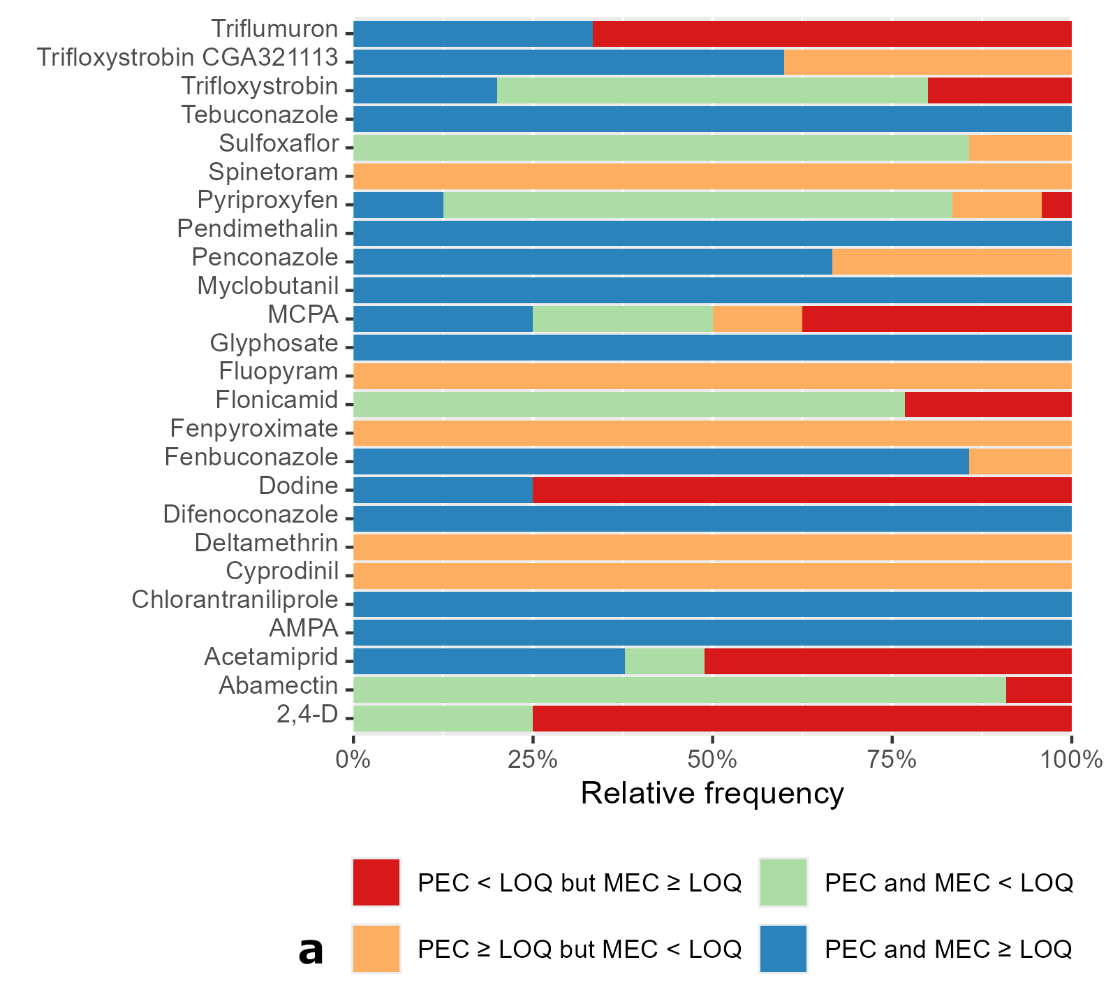 |
| --- |
| 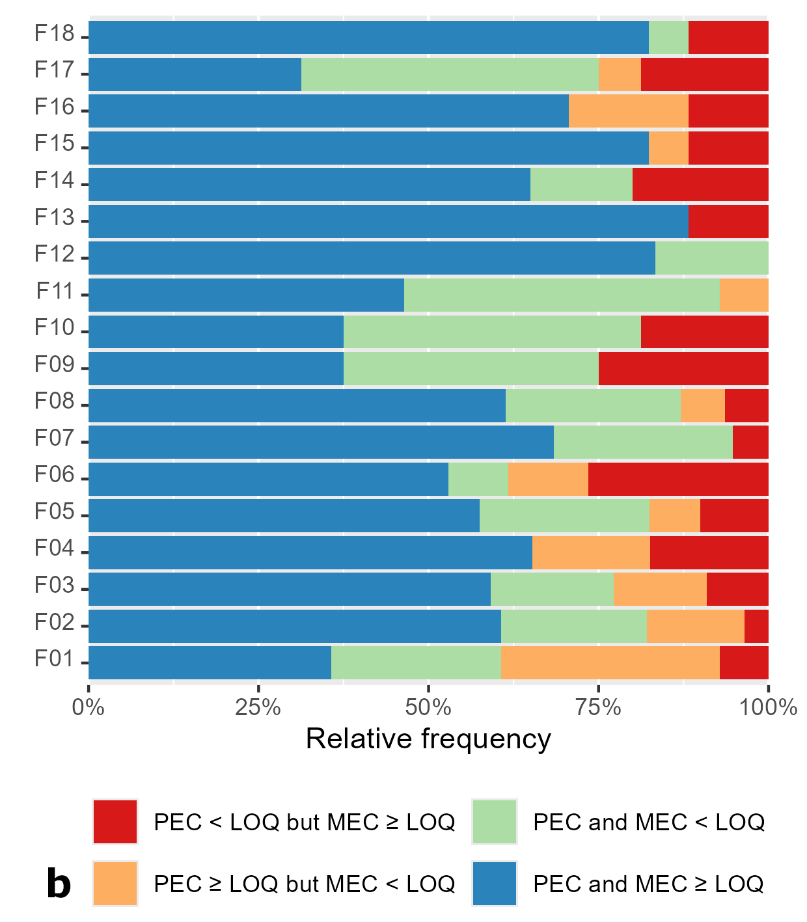 |
| **Fig. S2** Comparison of predicted and measured environmental concentrations (PEC and MEC) for active substances and metabolites (a) and for the studied orchards (b) according to the LOQ of each analysed active substance and metabolite. The results were divided in 4 categories: (1) PEC and MEC<LOQ, (2) PEC and MEC≥LOQ, (3) PEC<LOQ but MEC≥LOQ and (4) PEC≥LOQ but MEC<LOQ. The soil samples were collected in 2022, in each one of the 18 studied peach orchards located in the east-central Portuguese region of Beira Interior. |

| 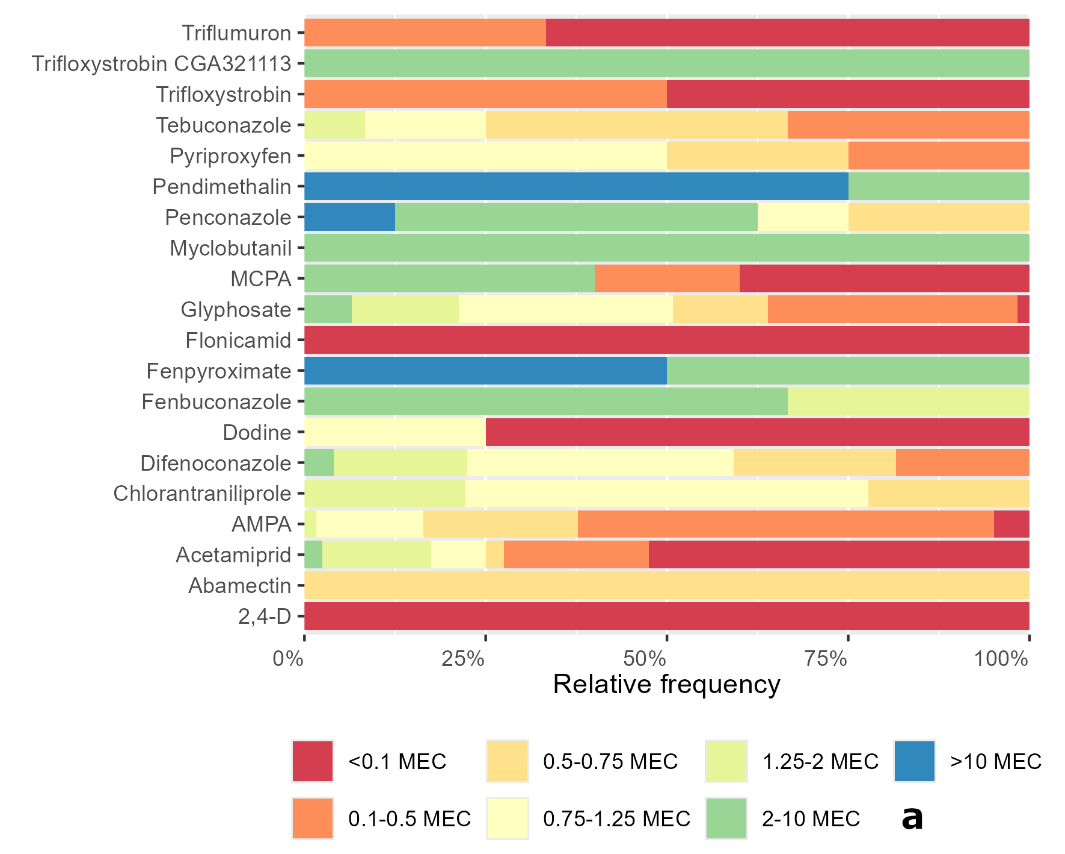 |
| --- |
| 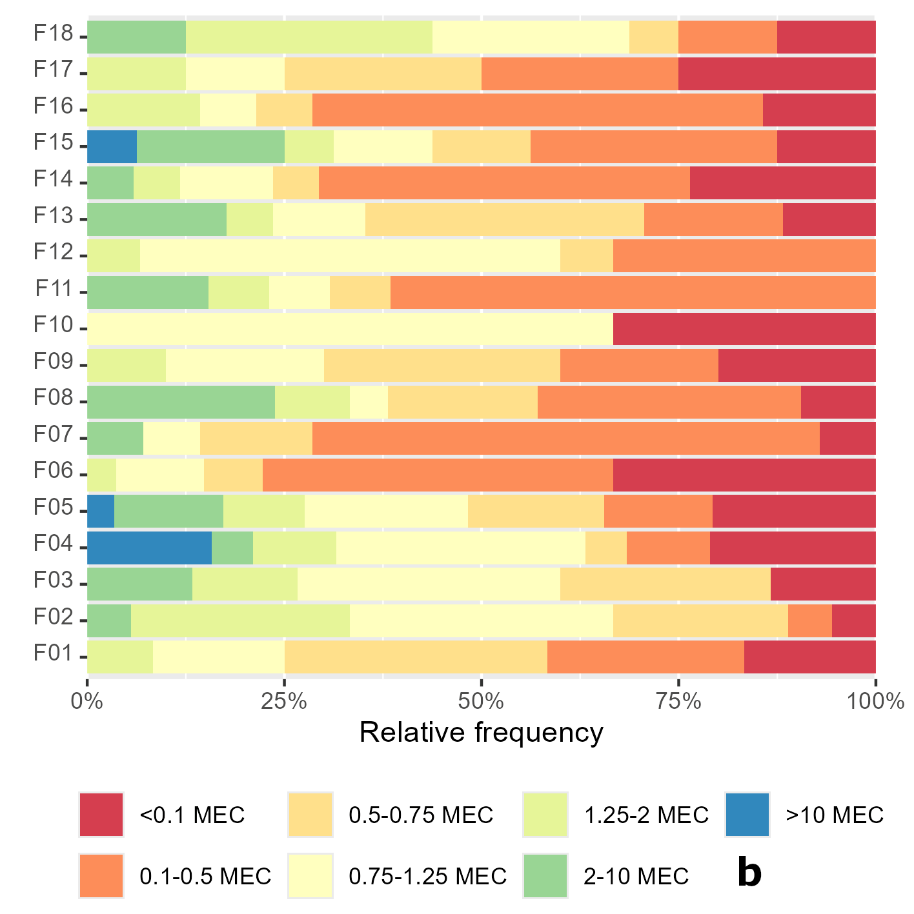 |
| **Fig. S3** Comparison between predicted and measured environmental concentrations (PEC and MEC) per compound (a) and per orchard (b). Only the results with MEC above LOQ were considered. The stacked bars represent the distribution of the cases by 7 categories, centred in the interval considered as a good correspondence between PEC and MEC, i.e., where PEC were between 0.75 and 1.25 MEC. The other 6 categories represent (1) mild deviations from MEC, i.e., PEC values that were between 0.50 and 0.75 of MEC or between 1.25 and 2.00 of MEC; (2) medium deviations, i.e., PEC values that were between 0.10 and 0.50 of MEC or 2.00 and 10.00 of MEC and (3) more extreme deviations, i.e., PEC values that were lower than 0.10 of MEC or higher than 10.00 of MEC. The soil samples were collected in 2022, in each one of the 18 studied peach orchards located in the east-central Portuguese region of Beira Interior. |

| 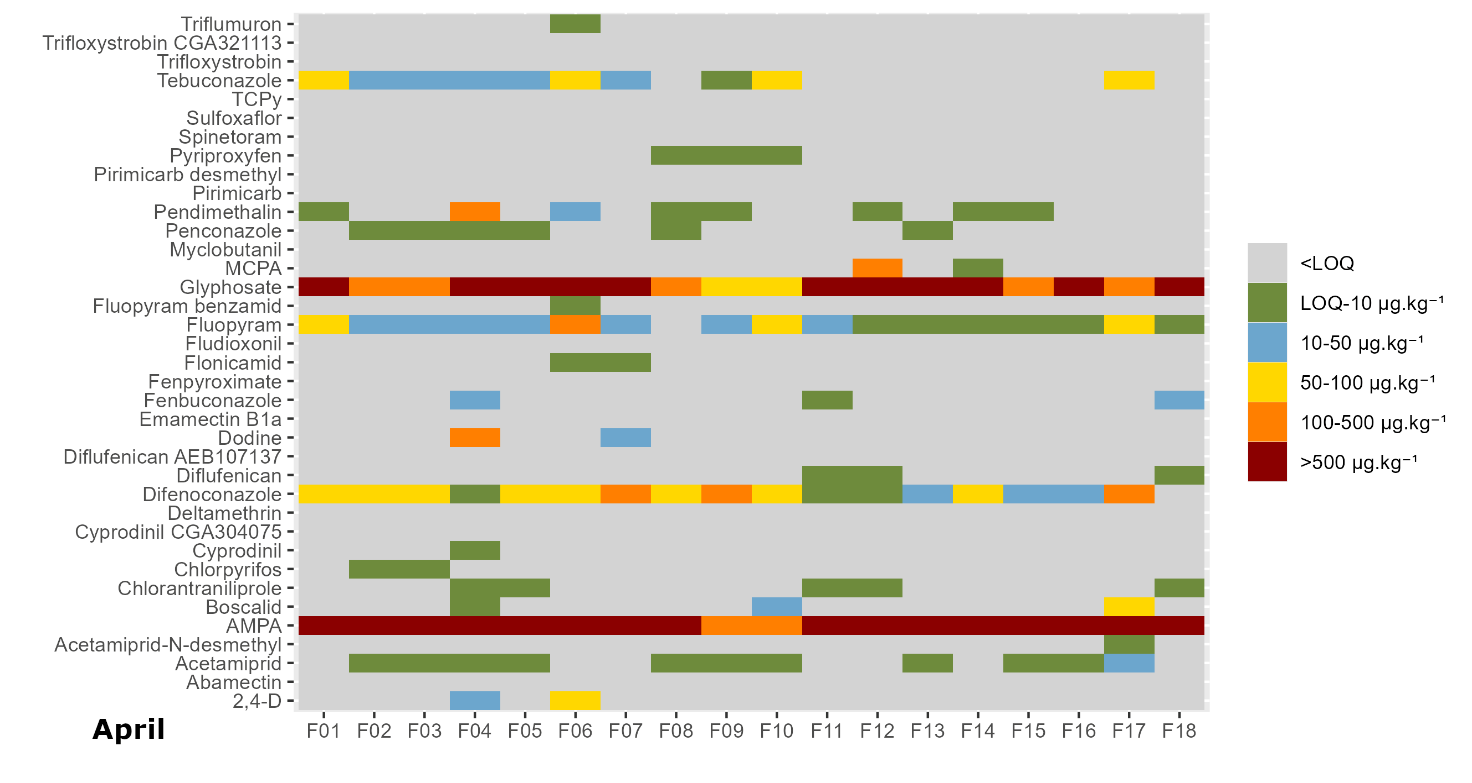 |
| --- |
| 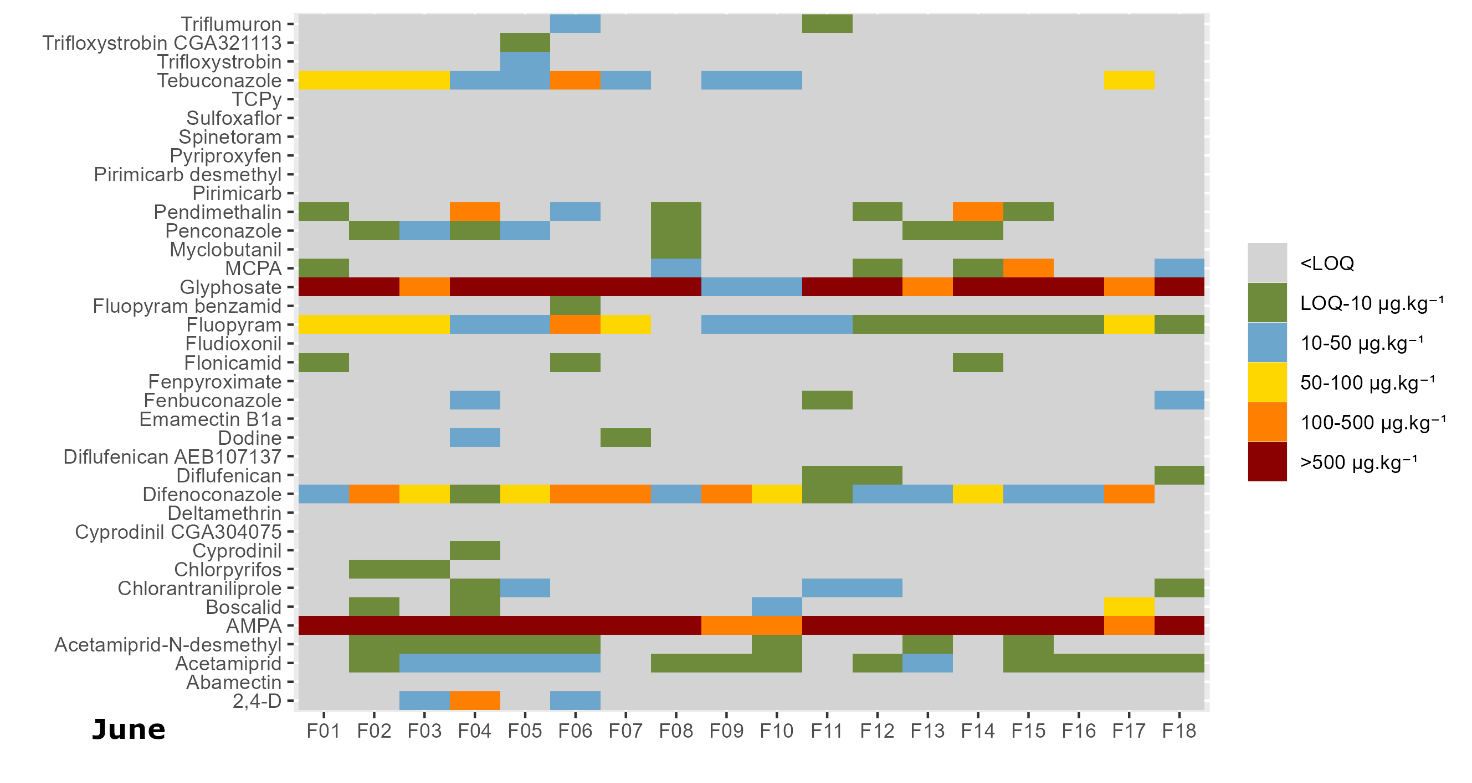 |
| **Fig. S4** Concentrations of active substances and metabolites in the soil samples collected in April and June 2022, in each one of the 18 studied peach orchards located in the east-central Portuguese region of Beira Interior. The rows represent the active substances and metabolites and the columns represent the orchards. The concentrations were divided into 6 categories, <LOQ, LOQ-10 µg.kg^‑1^, 10-50 µg.kg^-1^, 50-100 µg.kg^-1^, 100-500 µg.kg^-1^ and >500 µg.kg^-1^. The limit of quantification (LOQ) was different among the active substances and metabolites that were analysed and is shown in Table S1 (Supplementary material). |
| 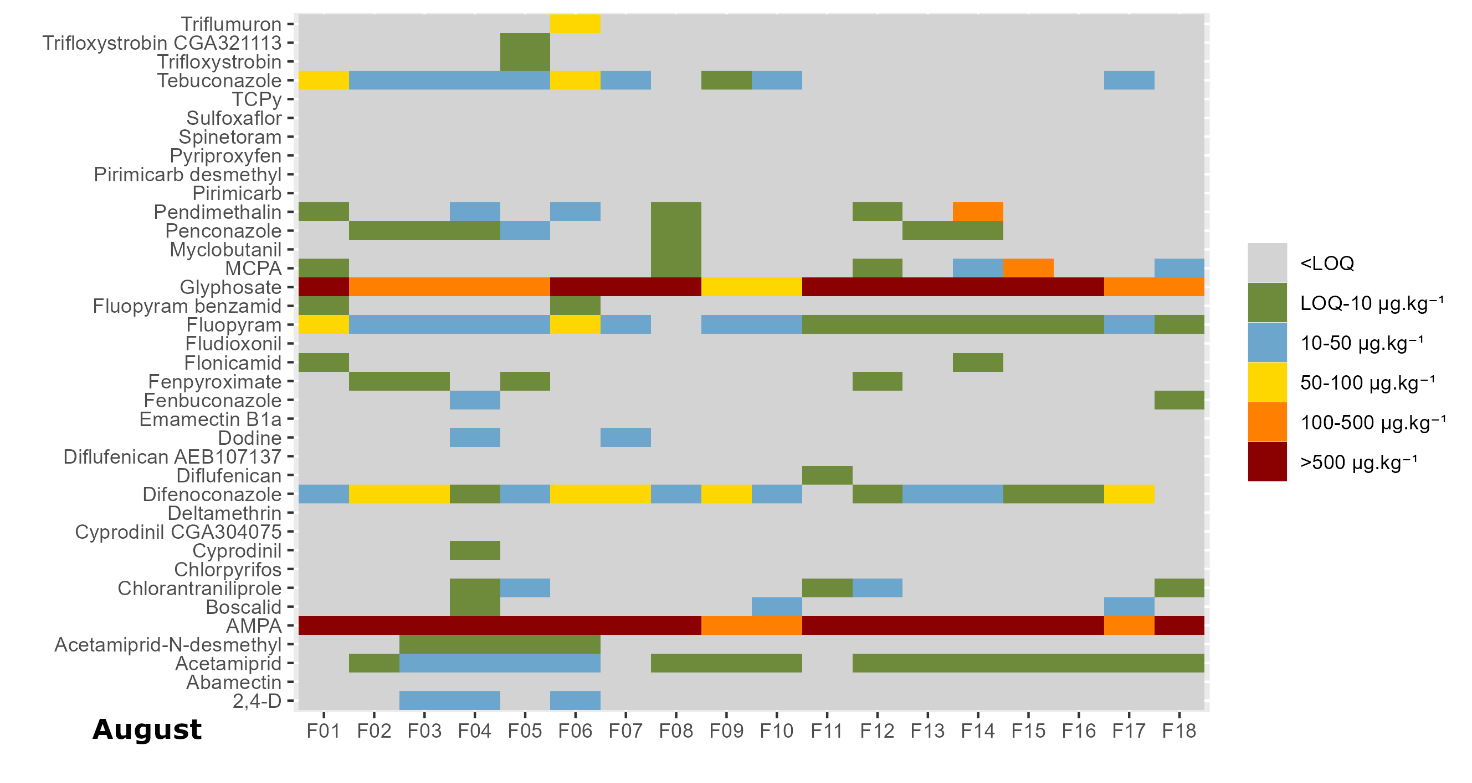 |
| **Fig. S4 (cont.)** Concentrations of active substances and metabolites in the soil samples collected in August 2022, in each one of the 18 studied peach orchards located in the east-central Portuguese region of Beira Interior. The rows represent the active substances and metabolites and the columns represent the orchards. The concentrations were divided into 6 categories, <LOQ, LOQ-10 µg.kg^‑1^, 10-50 µg.kg^-1^, 50-100 µg.kg^-1^, 100-500 µg.kg^-1^ and >500 µg.kg^-1^. The limit of quantification (LOQ) was different among the active substances and metabolites that were analysed and is shown in Table S1 (Supplementary material). |

**References**

EFSA, 2016a. Peer review of the pesticide risk assessment of the active substance acetamiprid. EFSA J. 14. https://doi.org/10.2903/j.efsa.2016.4610

EFSA, 2016b. Peer review of the pesticide risk assessment of the active substance pendimethalin. EFSA J. 14. https://doi.org/10.2903/j.efsa.2016.4420

EFSA, 2015. Conclusion on the peer review of the pesticide risk assessment of the active substance glyphosate. EFSA J. 13. https://doi.org/10.2903/j.efsa.2015.4302

EFSA, 2014. Conclusion on the peer review of the pesticide risk assessment of the active substance 2,4‐D. EFSA J. 12. https://doi.org/10.2903/j.efsa.2014.3812

EFSA, 2013. Conclusion on the peer review of the pesticide risk assessment of the active substance chlorantraniliprole. EFSA J. 11. https://doi.org/10.2903/j.efsa.2013.3143

EFSA, 2011a. Conclusion on the peer review of the pesticide risk assessment of the active substance difenoconazole. EFSA J. 9. https://doi.org/10.2903/j.efsa.2011.1967

EFSA, 2011b. Conclusion on the peer review of the pesticide risk assessment of the active substance triflumuron. EFSA J. 9, 1941. https://doi.org/10.2903/j.efsa.2011.1941

EFSA, 2010a. Conclusion on the peer review of the pesticide risk assessment of the active substance dodine. EFSA J. 8. https://doi.org/10.2903/j.efsa.2010.1631

EFSA, 2010b. Conclusion on the peer review of the pesticide risk assessment of the active substance fenbuconazole. EFSA J. 8. https://doi.org/10.2903/j.efsa.2010.1558

EFSA, 2010c. Conclusion on the peer review of the pesticide risk assessment of the active substance flonicamid. EFSA J. 8, 1445. https://doi.org/10.2903/j.efsa.2010.1445

EFSA, 2010d. Conclusion on the peer review of the pesticide risk assessment of the active substance myclobutanil. EFSA J. 8, 1682. https://doi.org/10.2903/j.efsa.2010.1682

EFSA, 2009. Conclusion regarding the peer review of the pesticide risk assessment of the active substance pyriproxyfen. EFSA J. 7. https://doi.org/10.2903/j.efsa.2009.336r

EFSA, 2008a. Conclusion regarding the peer review of the pesticide risk assessment of the active substance abamectin. EFSA J. 6. https://doi.org/10.2903/j.efsa.2008.147r

EFSA, 2008b. Conclusion regarding the peer review of the pesticide risk assessment of the active substance penconazole. EFSA J. 6. https://doi.org/10.2903/j.efsa.2008.175r

EFSA, 2008c. Conclusion regarding the peer review of the pesticide risk assessment of the active substance tebuconazole. EFSA J. 6. https://doi.org/10.2903/j.efsa.2008.176r

University of Hertfordshire, 2023. PPDB: Pesticide Properties DataBase [WWW Document]. URL http://sitem.herts.ac.uk/aeru/ppdb/ (accessed 8.23.24).
